# Supplementary material for: Reconciling ASPP-p53 binding mode discrepancies through an ensemble binding framework that bridges crystallography and NMR data
Source: PLoS Comput Biol. 2024 Feb 7;20(2):e1011519. doi: 10.1371/journal.pcbi.1011519 (PMC10878502; doi:10.1371/journal.pcbi.1011519)
Supplement: S1 Table — (PDF) [file pcbi.1011519.s001.pdf]

Table S1: Molecular dynamics simulations performed in this study.

Simulation Types	Cases	Starting Structures	Simulation Length
conventional all-atom MD	$p53_{DBD}$ (w/o Zn^{2+})	PDB 1YCS	500 ns
	$p53_{DBD}$ (w/ Zn^{2+})	PDB 1YCS	500 ns
	$p53_{DBD}$-ASPP2 (w/ Zn^{2+})	PDB 1YCS	1 μs $\times$ 3
	$p53_{DBD}$-iASPP (w/ Zn^{2+})	PDB 6RZ3	1 μs $\times$ 3
	$p53_{P\text{-}DBD\text{-}L}$-iASPP (w/ Zn^{2+})	PDB 1YCS + modelled p53 IDRs	1 μs $\times$ 3
	$p53_{P\text{-}DBD\text{-}L}$-iASPP (w/ Zn^{2+})	PDB 6RZ3 + modelled p53 IDRs	1 μs $\times$ 3
Martini CGMD	$p53_{DBD}$-ASPP2	PDB 1YCS	4 μs $\times$ 50
	$p53_{DBD}$-iASPP	PDB 6RZ3	4 μs $\times$ 50
	$p53_{P\text{-}DBD}$-ASPP2	PDB 1YCS + modelled Pro-domain	4 μs $\times$ 50
	$p53_{P\text{-}DBD}$-iASPP	PDB 6RZ3 + modelled Pro-domain	4 μs $\times$ 50
	$p53_{P\text{-}DBD\text{-}L}$-ASPP2	PDB 1YCS + modelled Pro-domain and linker	4 μs $\times$ 50
	$p53_{P\text{-}DBD\text{-}L}$-ASPP2	PDB 1YCS + Rigidified IDRs	4 μs $\times$ 50
	$p53_{P\text{-}DBD\text{-}L}$-iASPP	PDB 6RZ3 + modelled Pro-domain and linker	4 μs $\times$ 50
all-atom umbrella samplings	$p53_{DBD}$-ASPP2 (w/o Zn^{2+})	PDB 1YCS	10 ns/win $\times$ $\sim$30 win
	$p53_{DBD}$-ASPP2 (w Zn^{2+})	PDB 1YCS	10 ns/win $\times$ $\sim$30 win
	$p53_{DBD}$-iASPP (w/o Zn^{2+})	PDB 6RZ3	10 ns/win $\times$ $\sim$30 win
	$p53_{DBD}$-iASPP (w/ Zn^{2+})	PDB 6RZ3	10 ns/win $\times$ $\sim$30 win
	$p53_{DBD}$-ASPP2 (w Zn^{2+})	Martini CGMD sampled complexes	10 ns/win $\times$ $\sim$50 win $\times$ 20
	$p53_{DBD}$-iASPP (w/ Zn^{2+})	Martini CGMD sampled complexes	10 ns/win $\times$ $\sim$50 win $\times$ 20
