# Supplementary figures and images for: Reconciling ASPP-p53 binding mode discrepancies through an ensemble binding framework that bridges crystallography and NMR data
Source: PLoS Comput Biol. 2024 Feb 7;20(2):e1011519. doi: 10.1371/journal.pcbi.1011519 (PMC10878502; doi:10.1371/journal.pcbi.1011519)

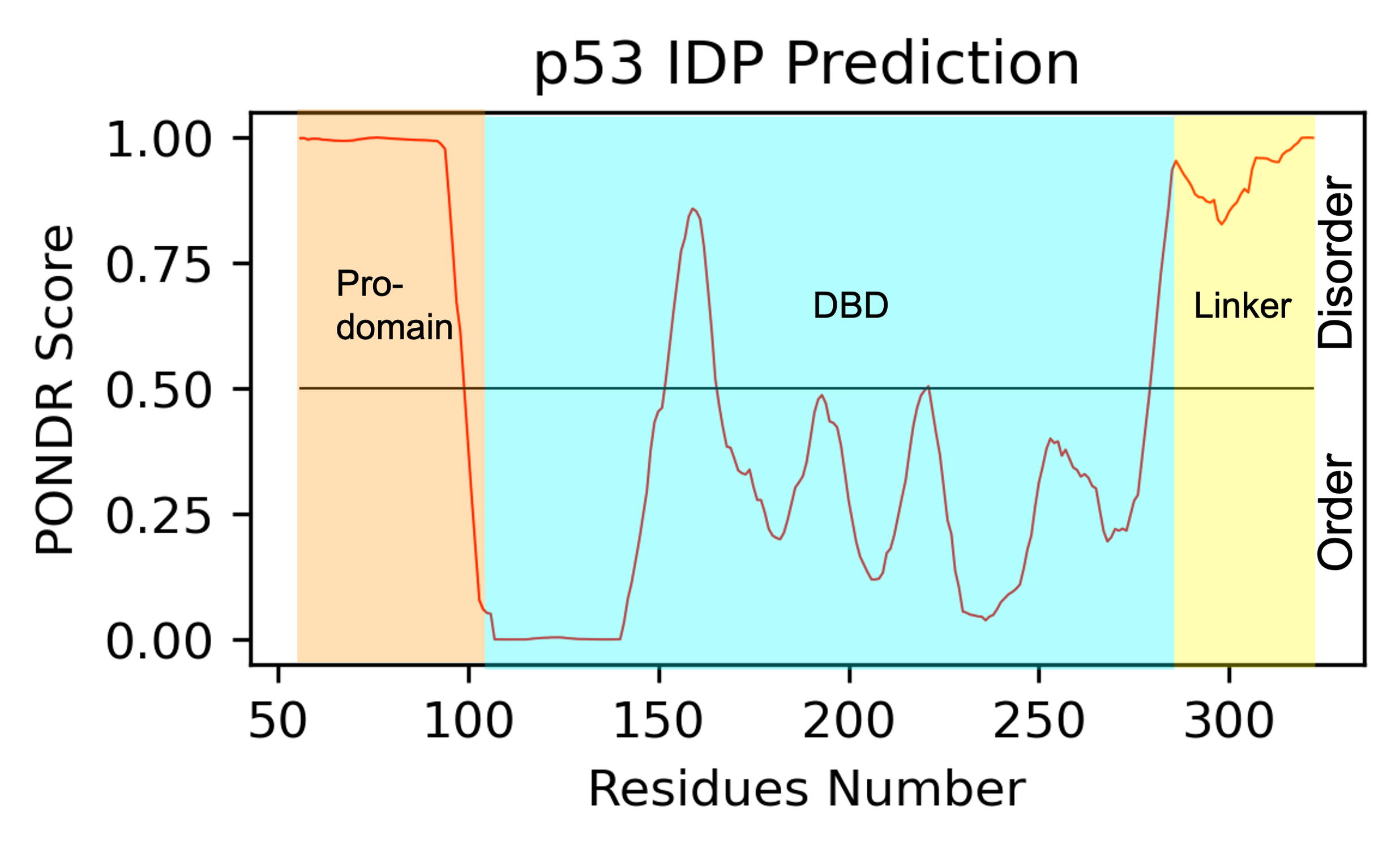

Supplement: S1 Fig — The Pro-domain and linker-domain flanking p53DBD are disordered. (TIF) [file pcbi.1011519.s002.tif]

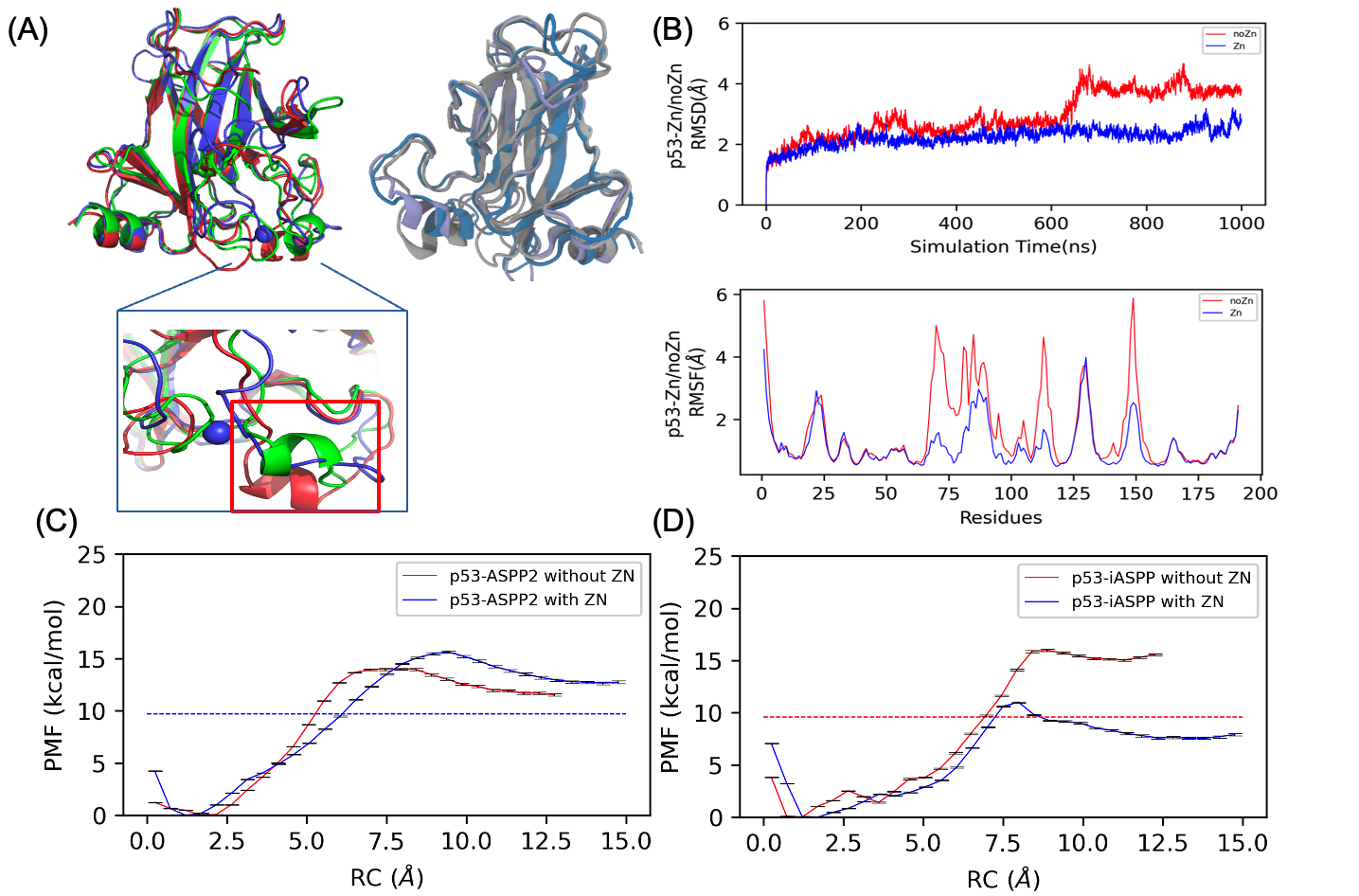

Supplement: S2 Fig — (A) Superimposing MD-sampled p53DBD conformations, w/ Zn2+ (blue) and w/o Zn2+ (red), on crystal structure (green). (B) RMSD and RMSF of p53DBD from MD simulations w/ and w/o Zn2+. (C-D) PMF of p53DBD disassociation from ASPP2 and iASPP w/ and w/o Zn2+. Errors bars were drawn in black, and were estimated by the built-in bootstrap error analysis of the WHAM program (num_MC_trials = 100). The relative small errors (∼0.1 kcal/mol) reflect that our 10 ns samplings per window in the umbrella sampling is sufficient. (TIF) [file pcbi.1011519.s003.tif]

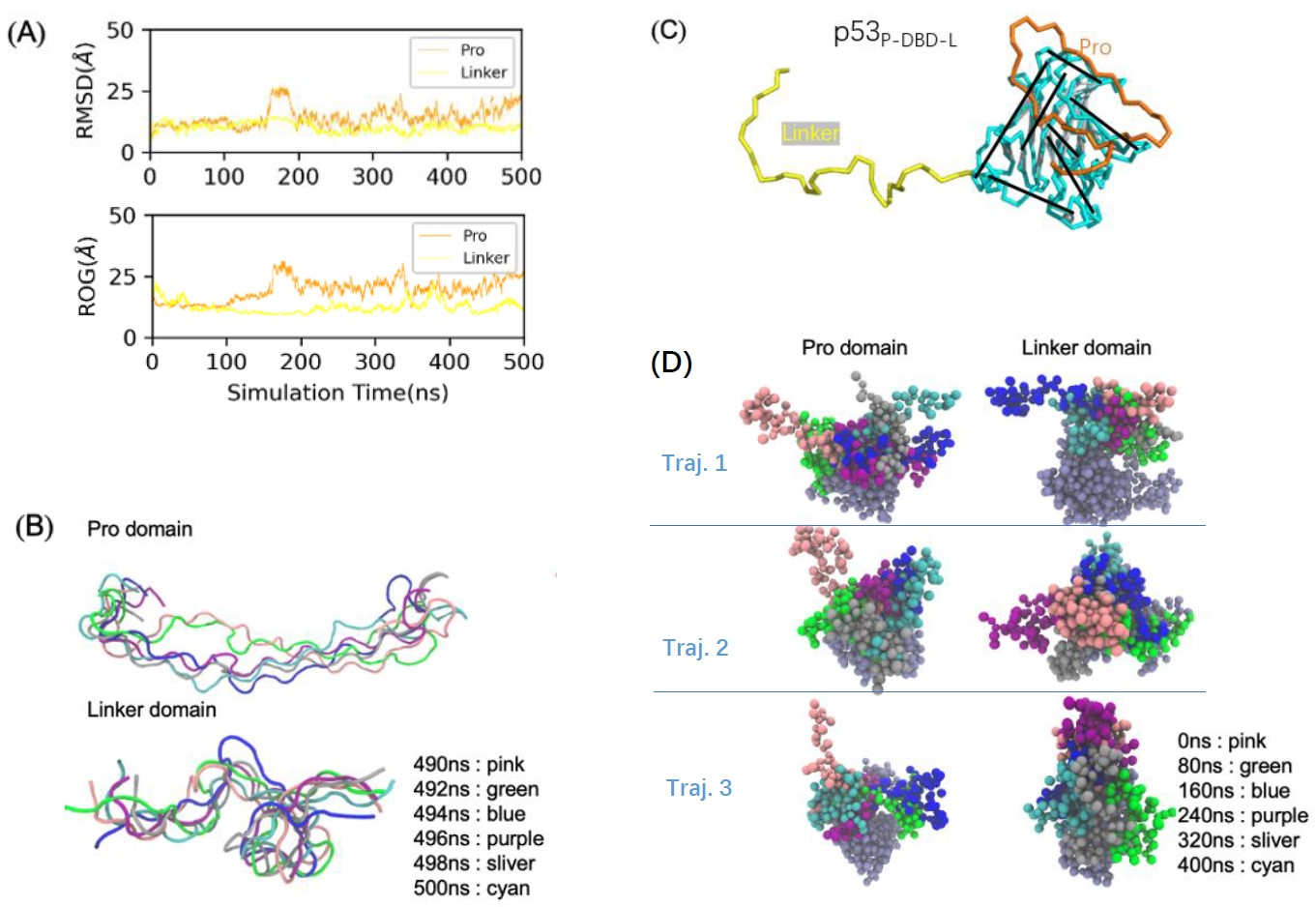

Supplement: S3 Fig — (A) RMSD and RoG (radius of gyration) of isolated p53 IDRs (Pro-domain and linker-domain) in 500 ns conventional all-atom MD. (B) Structures extracted from the last 10 ns trajectory of 500 ns MD at every 2 ns. (C) Schematic diagram showing the elastic networks that constrain only the DBD domain of p53P-DBD-L in the Martini CG simulations. (D) Selected Martini CGMD trajectories showing the highly dynamic IDRs of p53 prior to binding ASPP. During the 4 μs long Martini CG simulations, p53’s IDRs sampled various conformations before binding ASPP. (TIF) [file pcbi.1011519.s004.tif]

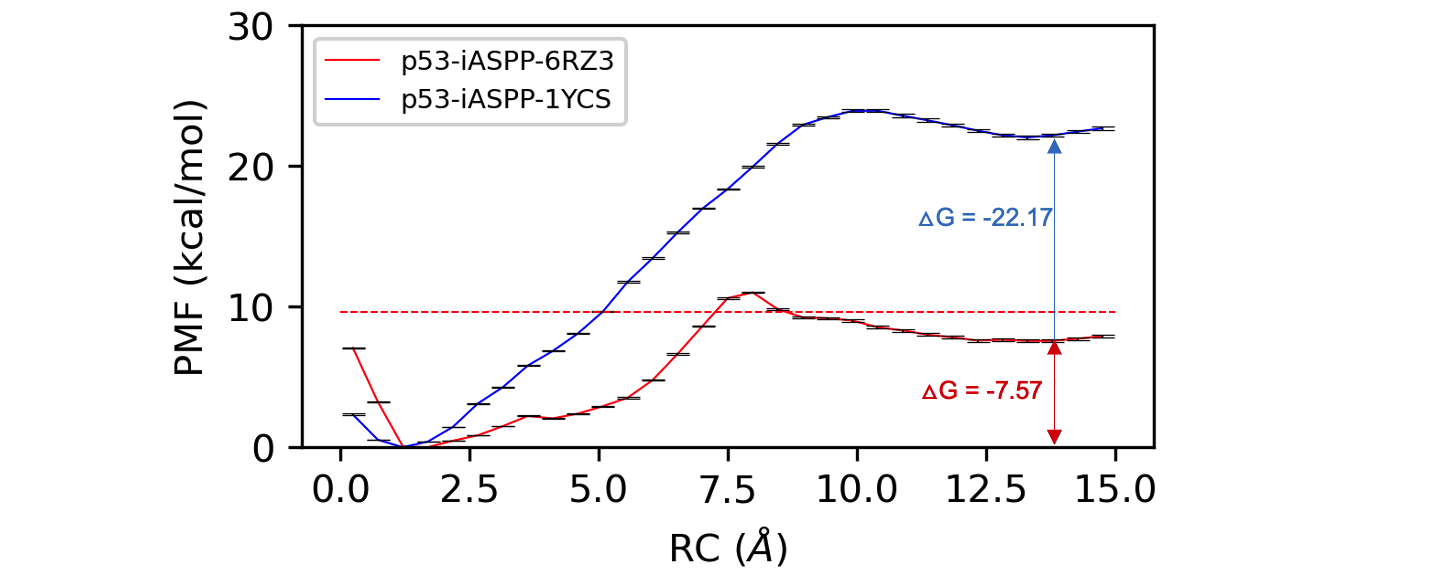

Supplement: S4 Fig — (TIF) [file pcbi.1011519.s005.tif]

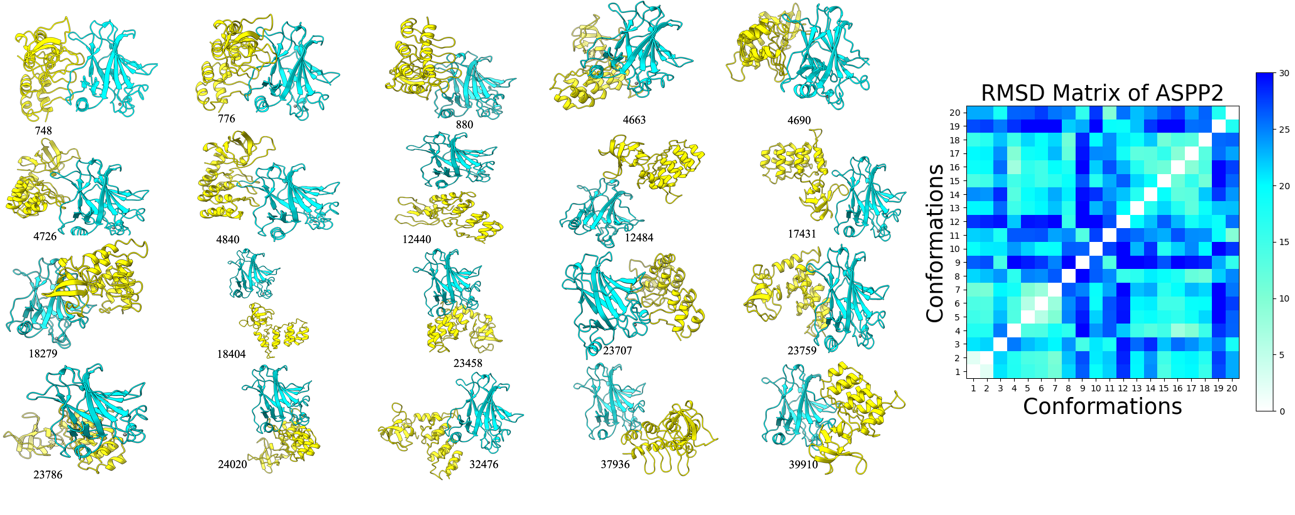

Supplement: S5 Fig — The pair-wise RMSDs of ASPP2 (after aligning on p53) were shown on the right to illustrate the structural difference of ASPP2. Procedure for selecting the complexes is: A center of mass (COM) distance criteria (<30 Å) and RMSD criteria (<50 Å, with respect to PDB 1YCS) were applied on the accumulated Martini trajectories to filter out frames that do not have p53 and ASPP contacted. The surviving trajectory frames were then aligned on p53DBD and were subject to following processes: ASPP protein densities around p53DBD were calculated using the grid command from the CPPTRAJ program. Regions have high relative density (> 0.6) were identified as the most probable binding patterns. The COM of ASPP was drawn around p53 for each frame. For those frames their COMs are located within the high density regions, they are identified as candidates. 20 frames were randomly picked out from the candidate pool. (TIF) [file pcbi.1011519.s006.tif]

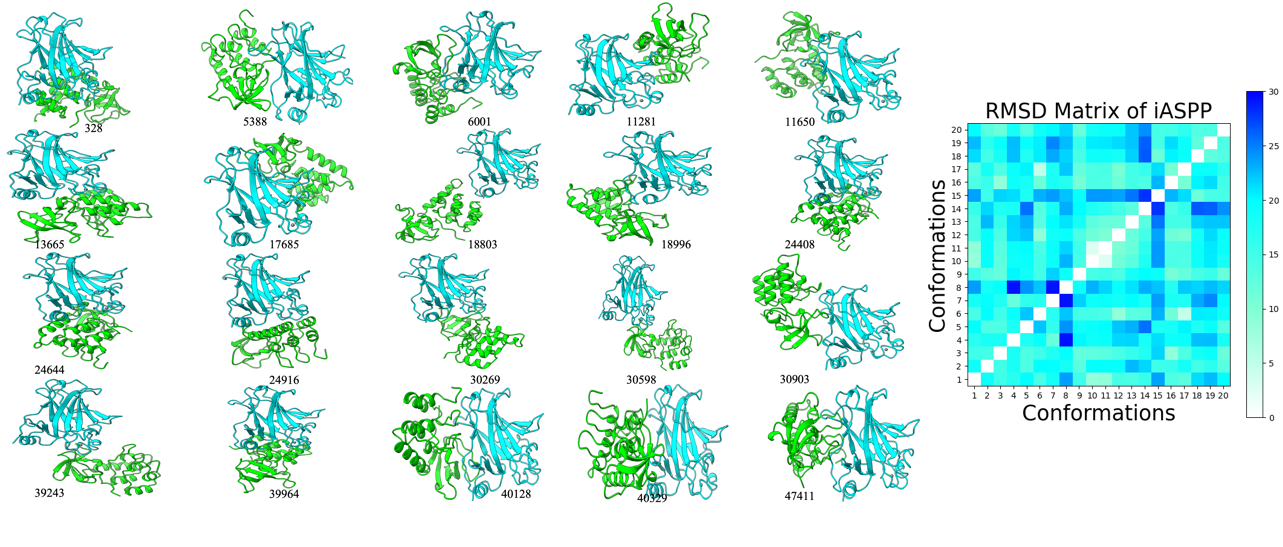

Supplement: S6 Fig — The pair-wise RMSDs of iASPP (after aligning on p53) were shown on the right to illustrate the structural difference of iASPP. (TIF) [file pcbi.1011519.s007.tif]

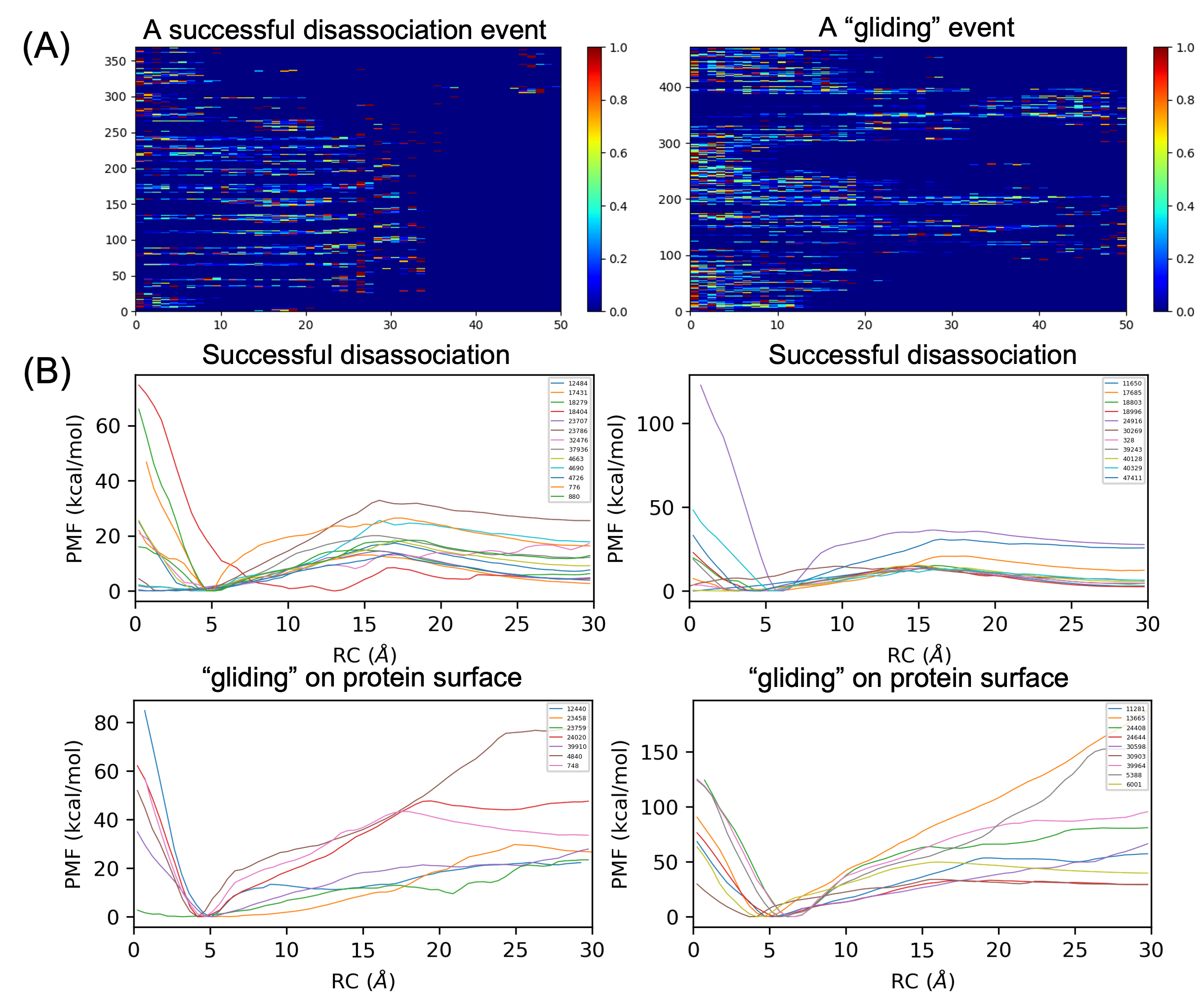

Supplement: S7 Fig — (A) Umbrella sampling using COM-COM distance as CV can lead to protein gliding on another protein’s surface instead of driving complex directly disassociate. We show that during the umbrella sampling, a successful disassociation has inter-protein contacts smoothly disappear as window number increases, indicating a clean one-way disassociation, while for a “gliding” event, newly formed inter-protein contacts are keep emerging as window number increases. (B-C) For the 20 representative Martini CGMD-sampled complexes for each ASPP protein, the PMF curves for successful dissociations (n = 13/20 for ASPP2, and n = 11/20 for iASPP), and for “glidings” are given. (TIF) [file pcbi.1011519.s008.tif]
