# Supplementary material for: Reconciling ASPP-p53 binding mode discrepancies through an ensemble binding framework that bridges crystallography and NMR data
Source: PLoS Comput Biol. 2024 Feb 7;20(2):e1011519. doi: 10.1371/journal.pcbi.1011519 (PMC10878502; doi:10.1371/journal.pcbi.1011519)
Supplement: Attachment — Submitted filename: Response.pdf [file pcbi.1011519.s009.pdf]

Reconciling ASPP-p53 Binding Mode Discrepancies through an Ensemble Binding Framework that Bridges Crystallography and NMR Data

Te Liu [1], Sichao Huang[1], Qian Zhang[1], Yu Xia[1],
Manjie Zhang[1*] and Bin Sun [1*]

[1] Research Center for Pharmacoinformatics, College of Pharmacy,
Harbin Medical University, Harbin 150081, China
* Corresponding author: binsun@hrbmu.edu.cn
zhangmj454@nenu.edu.cn

December 11, 2023

To assist the reviewers, we provide below a manuscript version that marks our modifications in blue and replaced text in red. In each response, we provide a hypertext link to the page number where changes were made.

0.1 Peer Reviewer #1:

Major issues:

1. *For the simulations of the IDRs alone, what method was used to determine that sampling was sufficient? 500 ns does not seem long enough to capture the structural ensemble of a 48-residue disordered sequence without employing an enhanced sampling method.*
   - **Response**
     We appreciate the reviewer's question, and we acknowledge that a 500 ns conventional molecular dynamics (cMD) simulation cannot thoroughly explore the conformational space of an IDR of this size. However, it is essential to clarify that the primary objective of the 500 ns MD is not to comprehensively explore the entire IDR ensemble but rather to generate a relatively stable structure for subsequent simulations.
     We have confirmed that this extended simulation duration is adequate to achieve a 'local minima' conformation of the IDR, as demonstrated by the consistent RMSD and radius of gyration (RoG) of the IDRs (see Fig. S3). In the subsequent molecular dynamics simulations, where the IDRs are attached to p53's DBD, whether through all-atom conventional MD or Martini CGMD, these IDRs continue to exhibit highly dynamic natures (see Fig. 2 and Fig. S3D).
     Therefore, we firmly believe that the 500 ns cMD serves its purpose well by providing a solid starting structure. Conducting longer cMD simulations to explore the complete conformational space would not yield substantial additional value to our results. We have revised the text to elaborate on our rationale behind choosing a 500 ns cMD and how it aligns with our research objectives. **See tag R1Q1a on Page 12, Line 519** and **See tag R1Q1b on Page 12, Line 534**
2. *Figure captions in general should include more information about what is being shown in the figures.*
   - **Response**
     Thanks for the suggestions, we have added more detailed information in the figure captions.
3. *Figure 3a and b: While the results somewhat overlap for NMR and CG simulations, it is not clear how good the agreement is, since there are some bars which don't overlap and it is hard to see where the differences are. It would be helpful to use a quantitative metric to capture the overall agreement and have a control such as comparing the NMR data to the crystal structure to show that these interfaces agree better with the NMR data than the crystal structure interface. Additionally, error bars could be constructed to quantify the variation between CG simulations to give a sense of the precision of the simulation results.*
   - **Response**
     Thank you for the suggestions, and we agree that employing a quantitative metric to assess the overlap between Martini CG-sampled residue contact frequency and NMR-measured chemical shift changes is more objective and informative. However, we recognize the challenge of finding an accurate metric, as these two quantities, while closely related in annotating PPI surface residues, are inherently different.
     Nonetheless, we addressed this challenge by quantifying the deviations between Martini CG results and NMR chemical shifts using the Root Mean Square Error (RMSE) metric: $RMSE = \sqrt{\sum_i (CG_i - NMR_i)^2/N}$, where NMR_i and CG_i are NMR data and Martini normalized contact frequency (average value after bootstrapping) for all ASPP residues. We reported RMSE values of 0.069 and 0.059 for iASPP and ASPP2, respectively. Our interpretation suggests that the simulated results and NMR data exhibit moderate deviation, with more noticeable differences for iASPP. However, upon mapping the CG and NMR data onto ASPP proteins (see Figure 3B), the overall interfacial regions identified by NMR are qualitatively captured by Martini CGs.
     We appreciate the reviewer's suggestion that using the crystal structure binding mode as a control is more informative. Yet, comparing crystal structure interfacial residues with NMR data proves challenging because these residues lack scalable quantities for comparison with NMR/Martini data. Instead, we marked the interfacial residues observed from crystal structures with "*" markers, employing different styles for distinction.
     Furthermore, we have recalculated the Martini CG residue contact frequency through bootstrapping to estimate statistical uncertainty. The means and standard errors of means were determined via a bootstrapping procedure, involving 10,000 resamplings

(sample size equals 50) from the original 50 simulation replicas. We have added these information in the main text **See tag R1Q3a on Page 15, Line 626**.

4. *Figure 4b: It is surprising that the binding plots for ASPP2 and iASPP look so similar especially since the RMSD on the y-axis is with respect to a different crystal structure in each case. This does not seem consistent with the differences between the binding that are shown in Figure 4 c. Perhaps this is related to a distinction between the encounter complex and the fully bound state, but this discrepancy should be explained. Additionally, the caption should mention whether it is free energy or frequency that is being plotted and what the color scale means.*
   - **Response**
     We regret to inform you that we mistakenly employed the same projection plot in Fig. 4B, leading to confusion. We have rectified this error in Fig. 4B. Nevertheless, it is important to note that even in the corrected panel, the binding plots between iASPP and ASPP2 still exhibit similarity. We attribute this similarity to the inherent correlation between the two projection axes: the inter-protein distance and RMSD. As these axes are correlated and not orthogonal, they lack optimal differentiating power. However, it is worth emphasizing that using these axes enables direct observation of overall bound complexes and whether these complexes align with the crystal structures. In this context, we believe that the projection plots remain appropriate for their intended purpose. We have revised the text accordingly to convey these points, and we have included descriptions for the colorbars. **See tag R1Q4 on Page 17, Line 675.**
5. *Figure 4d: It would be helpful to include the delta G values from the PMFs starting from the crystal structures for comparison. The caption also needs to be expanded to explain what the black dashed lines are and the line labeled "ns".*
   - **Response**
     Thanks for the suggestion.
     Originally, the "ns" indicated no significance. We have redrawn Fig. 4D to incorporate crystal structure-based ΔGs and the pairwise t-test between experimental ΔGs and various calculated ΔGs.
     In the revised Fig. 4D, we present the crystal structure-based ΔGs alongside the results of pairwise t-tests comparing experimental ΔGs with those calculated by different methods. This addition provides a comprehensive and clearer representation of the data, allowing for a more robust interpretation of the significance levels in the binding energetics.
     We appreciate the reviewer's keen eye in pointing out the need for improvement in this aspect, and we believe that these modifications enhance the clarity and rigor of our analysis. The text has been updated accordingly to reflect these changes.
6. *Page 9: The number of weaker binders for ASPP2 and iASPP in Figure 4d is actually about the same. ASPP2 seems to have more tighter binders. A t-test to see if there is a statistically significant difference between the binding free energies for the crystal structures and those from the CG simulation-generated poses would help show that the simulation poses are actually more consistent with experimental binding data than crystal structures.*
   - **Response**
     Thanks for the insightful comments. As suggested by the reviewer, we have performed the t-test between CG simulation-generated poses and crystal structures for iASPP and ASPP2, and t-test between various calculated binding free energies and experimental affinities (see the new Fig. 4D). We briefly summarized the observations here: For ASPP2, for which the reported crystallography binding mode is consistent with solution NMR data, the crystal structure based ΔG and CG-simulated poses based ΔG are not significantly different ($p = 0.284$). For iASPP, while the reported crystal structure (PDB 6RZ3) also does not show significant ΔG difference with that from Martini simulations ($p = 0.438$), its another NMR-suggested binding mode (PDB 1YCS) is significantly different from the Martini ΔG ($p = 0.0008$). Importantly, for both ASPP2 and iASPP, ΔGs calculated from crystal structures are significantly different from the experimentally-measured binding affinities. However, the ΔGs based on Martini simulation poses are not statistically different from experimental values, with p=0.623 and 0.781 for ASPP2, and iAPP, respectively. These data suggest that using Martini CGMD sampled poses can better capture the experimentally measured binding affinities for both ASPP2 and iASPP. We have reported these t-test results in the main text. **See tag R1Q6 on Page 17, Line 700**
7. *Additionally, it would be interesting to know if there was a free energy difference for the two different iASPP poses, which are presumably included in the different binding complexes sampled in Figure 4d.*

- **Response**
  Thanks for the question. We actually have performed the PMF calculations for the two iASPP poses, starting from PDB 1YCS and PDB 6RZ3, respectively (Fig. S4). These two poses do show ΔG difference of 14.6 kcal/mol. Thus these two resemble the weak and strong binders we selected for iASSP from Martini CGMD-sampled complexes. **See tag R1Q7 on Page 17, Line 697**

8. *Figure 5a: The encounter time measurement appears to be based on any contact between p53 and ASPP, but this leads to inconsistency because the simulations with the p53 IDRs have more residues that p53 could use to interact with the ASPP. To determine whether the IDRs are actually affecting binding kinetics, just the contacts between p53-DBD and ASPP could be used even in the simulations with the IDRs present. This would allow a consistent comparison between the simulations. Additionally error bars for this plot calculated based on variation between the independent simulations would be helpful, or a t-test to show there is a significant difference.*
   - **Response**
     We thank the reviewer's insightful comments to improve the rigorousness and clarity of our analysis on IDR-related binding kinetics. Firstly, we would like to clarify our way of calculating the "encounter time" is based on the formation of first inter-protein contact, which we recognized that it does not represent exactly the encounter complex, and hence we have changed the y-axis label of Fig5A as "first contact formation time", and would like to use this quantity to approximate the real encounter complex formation time. We also agree that the greatly shortened "first contact formation time" for IDR-containing constructs may be just due to larger size of the p53 constructs that allow ASPP to more easily interact, rather than is the dynamic nature of IDRs that accelerates the binding. This question is also pertain to the reviewer's 14th question, in which reviewer has pointed out that our explanations of the accelerated binding kinetics using the "fly-casting" mechanism is not well-supported by current data.
     We agree with both the comments, and to gain solid understandings, we did the following two calculations, as per the reviewer suggested:
     - Calculated the first contact formation time between p53 DBD domain and ASPP even if p53 IDRs are present.
     - Performed one additional set of Martini CGMD simulations in which p53 IDRs were rigidified via constraints. This was to validate if the dynamic nature of the IDRs affect the binding rates.

     PS: We are sorry to find that in the original Fig5A, we labeled the unit as nanosecond (ns) but was showing the frame numbers. We have converted to "ns" by multiplying 4ns/frame in the latest Fig5A.
     Our new observations are: as shown in Fig. 5C, counting only p53's DBD domain interacting with ASPP still shows comparable first contact formation time as counting the whole p53's constructs. A negative control in which treating IDRs as rigid motifs reverse the binding accelerating phenomena, resulting in a much slower binding for the $p53_{\text{P-DBD-L}}$. These data suggest that at local structural level, the p53's DBD, regardless the presence of IDRs, is the predominant domain to first interact with ASPPs. More importantly, the shortened first contact formation time, thereby the accelerated binding kinetics of $p53_{\text{P-DBD-L}}$ relative to $p53_{\text{DBD}}$ are caused by the dynamics of IDRs. We believe that these additional calculations as well as the reviewers insightful comments have helped to clarified our presentations on the IDR-accelerated binding kinetics. We have revised to main text to reflect this point **See tag Q1Q8a on Page 19, Line 724** and **See tag R1Q8b on Page 22, Line 817**

9. *Figure 5b: The number of contacts with the DBD without the IDRs there should be shown on the plot for comparison. As it is, it is hard to see that the IDRs change the amount of contact with the DBD for iASPP.*
   - **Response**
     We have added the average contact between DBD and ASPP without IDR presence based on the ASPP- Martini trajectories. In the new Fig. 5B, it shows that p53 DBD's interactions with both ASPPs are attenuated when p53 IDRs are present, exhibiting a clear sign of p53's IDRs in tuning the binding patterns of the PPI. We have included this point in the main text **See tag R1Q9 on Page 19, Line 743**

10. *Fig 5C: The sketch plot is helpful for seeing that the binding pose changes, but all structural information is obscured by the choice of reaction coordinates. It would be helpful to have an example pose shown for each of the highly sampled states. It would be informative to see how the most common binding poses with the IDRs present compare to the binding poses discussed above in Figure 4b&c.*
    - **Response**
      We appreciate the reviewer's comments, and in response to the suggestion, we have

included representative complexes to guide the interpretation of sketch-map plots. In our analysis, we have introduced the terms "prime" and "secondary" binding modes to distinguish between the most highly-sampled states and the less sampled but still considerably populated states (if present), respectively.
Consistent with our interpretations on sketch-map, we show that, in the absence of IDRs, although the prime binding modes of ASPP2 and iASPP towards $p53_{\mathrm{DBD}}$ are different, their secondary binding modes have similar complexes. Adding IDRs diminishes the overlap and leaves the utterly unlike prime binding modes of ASPP2 and iASPP. We have added these points **See tag R1Q10 on Page 21, Line 761**

11. *Page 10, paragraph 1: The discussion of binding kinetics is confusing. It is stated that the p53 construct with the IDRs binds more quickly in the CG simulations, but then that the addition of IDRs does not significantly alter binding kinetics. The discrepancy here should be explained.*

  - **Response**
    Thanks for pointing out this vague point. We intended to say that, while including p53 IDRs greatly accelerates the protein binding kinetics compared to the no IDR cases, the extents of accelerations are comparable for both ASPP2 and iASPP. Thus IDRs do not differentiate p53's binding patterns towards ASPP2 and iASPP kinetically. We have revised the main text **See tag R1Q11 on Page 19, Line 739**

12. *Based on the discussion it seems that the authors are making a distinction between the protein-protein interactions that they observe in their simulations which are fully bound and those that are part of the encounter complex, during the binding process. However, it is not clear how they are distinguishing between these two states and which results are focused on the bound state or the encounter complex in figures 3, 4, and 5.*

  - **Response**
    This is a very insightful question. As the reviewer has pointed out, throughout the manuscript, except for one paragraph in the Discussion section, we implicitly treated the Martini CGMD simulated complexes as fully bound states. Therefore, for Figures 3, 4, and 5, the presented data and discussions are in the context of the 'fully bound state.'
    In the second-to-last paragraph of the Discussion section, we did indeed shift the focus to the 'encounter complex' but inadvertently omitted providing the context within which the encounter complex was defined.
    We have now revised the paragraph as follows:
    Although we implicitly treated the Martini CGMD sampled complexes as 'fully bound complexes' in previous sections, a closer examination of the time-dependent binding process reveals that these simulated complexes were experiencing different degrees of refolding (see Fig. 6). While in some cases, the ASPP and p53 did not refold (e.g., bind and stick together), in most simulation trajectories, these two proteins underwent, and were likely to continue, refolding events. We speculate that over a much longer time-scale, most of our Martini CGMD sampled complexes would continue to refold and converge to fewer binding modes that are more biologically relevant.
    From this perspective, the Martini CGMD complexes can be loosely treated as an 'encounter complex' that would continue their journey to the destined native modes. In this context, our Martini CGMD amounts to completing the diffusion of protein partners to form the encounter complexes, which await rearrangements to reach the native Protein-Protein Interaction (PPI) state, such as rolling on another protein's surface. **See tag R1Q12 on Page 22, Line 828**

13. *Page 12, paragraph 2: In the discussion it says that the KDs are in the uM range, but earlier it says they are between 20 and 30 nM, not near the 100 uM threshold. This discrepancy should be explained.*

  - **Response**
    We are sorry for not assigning the various experimental K_D values to their respective systems, as those K_Ds were reported by different groups on various p53 constructs. The nanomolar range 20 to 30 nM K_Ds are for the folded p53's binding ($p53_{\mathrm{DBD}}$) to ASPP2/iASPP [Ref7] measured by ITC experiment, while micromolar ranges were reported for $p53_{\mathrm{P\text{-}DBD}}$'s binding towards ASPP2/iASPP. Specifically, Ahn *et al*[Ref4] used NMR titration experiments to report that $p53_{\mathrm{P\text{-}DBD}}$ has K_Ds of 76.7 ± 10 μM and 1.3 ± 0.2 μM towards iASPP and ASPP2, respectively. In the discussion, we want to refer to the cases where IDRs were attached to p53, the binding affinities can be close to the 100 μM threshold, which can cause uncertainty in assigning accurate binding mode. We have revised the discussion to be more precise regarding the statements. **See tag R1Q13 on Page 22, Line 789**

14. *Page 12, paragraph 4: In the discussion the authors argue that this study demonstrates the fly-casting method because the IDRs accelerate binding. However, it is not clear that the increase in binding rate would be significant at relevant concentrations, and may be more due to the size of the IDRs than the fact that they are disordered. The difference in the interaction contacts due to the addition of the IDRs is more interesting, but is hard to interpret due to the opacity of the sketch-map presentation of the data. Additionally, the authors state that these states represent the encounter complex but it is not clear how they are defining that.*

    - **Response**
      Please refer to our responses to earlier questions for detailed explanations. In summary, the data presented in Fig. 5A, specifically the negative control where IDRs are treated as rigid motifs, supports the conclusion that it is the dynamic nature of the IDRs, rather than their large size introduced into the system, that accelerates the binding process. Additionally, the inclusion of representative sketch-map structures has been provided to emphasize the specific inter-protein contacts, further supporting the notion that the presence of IDRs fine-tunes these inter-protein interactions.
      During the preparation of the revision, and with gratitude for the reviewer's expertise in IDRs, we recognized that our previous usage of the term "encounter complex" was not rigorously accurate. We would like to clarify that, particularly in this context, we are referring simply to the Martini simulated complexes and not the more specific term "encounter complex." This correction has been implemented throughout the manuscript to ensure precision and clarity in our terminology**See tag R1Q14c on Page 22, Line 819**

15. *Page 12, paragraph 5: The argument around IDRs changing the "effective concentration" of the binding partner is unclear. The authors may be referring to the idea that the protein will be more likely to stay in contact with the binding partner instead of diffusing away because the IDRs will facilitate contact with the binding partner, but this is not sufficiently explained. Where the encounter complex fits in to the data presented is also unclear.*

    - **Response**
      We wholeheartedly agree with the reviewer's comments regarding effective concentration. As the reviewer accurately speculated, the original concept of IDR-related effective concentration stems from the idea that IDRs can function as tethers, holding proteins together and preventing them from diffusing away. This, in turn, increases the likelihood of substrates being in the vicinity of the protein active site [Valen et al., Biophys. J., 2009, 96, 1275–1292].
      Initially, we used the term 'effective concentration' with the intention of referring to the size of candidate pools that can successfully refold. However, we now recognize that this usage of the term is misleading. Consequently, we have deleted it and revised the text as follows:
      Our aim was to convey the idea that, assuming our simulated p53-ASPP complexes need to refold to attain biologically relevant binding modes, and given our demonstrated finding that p53's IDRs can fine-tune protein interaction patterns, it is likely that the addition of IDRs can alter the portions of simulated complexes that successfully reach the desired states. This is because the refolding process is conditional, occurring on subsets of encounter complexes that satisfy specific criteria such as shape complementarity [Ref55]. In this context, the inclusion of IDRs in p53 increases the size of the candidate pool from which more complexes can potentially refold to the native PPI state. **See tag R1Q15 on Page 22, Line 826**

Minor:

1. *Abstract: Unclear what "the ensemble-average inter-protein contacting residues and NMR-detected interfacial residues align well with ASPP proteins" means. Do you mean that these groups of residues align well between the two ASPP proteins?*

    - **Response**
      Sorry for this unclear expression. We intended to say that the calculated PPI interfaces generally match the NMR-detected PPI interfaces, when rendered on the ASPP proteins. We have changed the sentence as "We showed that the ensemble-average inter-protein contacting residues and NMR-detected interfacial residues qualitatively overlap on ASPP proteins"

2. *Page 3, paragraph 1: "Consequence of p53 activation is majorly cell apoptosis, but can also cause" should be "The main consequence of p53 is cell apoptosis, but it can also cause"*

    - **Response**
      Done

3. *Page 3, paragraph 1: "are much wanted yet are obscured by lacking of definite structural characterizations" should be rephrased.*

- **Response**
  Thanks for the suggestion. We have revised the sentence: "Given the critical role p53 plays in tumors, mechanical insights into the reversal regulatory effects of ASSP2 and iASPP on p53 are of great importance for potential therapeutic developments. However, understanding is obscured, in part, due to controversial binding modes that have been reported on these PPIs".

4. *It is not mentioned if the particle-mesh Ewald method is used to handle long-range electrostatic interactions in the all-atom simulations.*

   - **Response**
     We verified that the PME method was used in all-atom simulations, and added this information in the Method section **See tag R1Qm4 on Page 11, Line 504**

5. *Figure 3: The layout of the figure is confusing. It was not clear at first that each panel contains a top and bottom part. A border around each panel might help. For panels a & b, the caption should read "Normalized contact frequency" rather than "Normal contact"*

   - **Response**
     Thanks for your suggestion and careful review. We have added light-colored outlines for each panel or have increased the gap between panels to make them more separable to improve figure readability. We have also changed the caption accordingly.

6. *Figure 3 caption: Each panel should be described in the caption. The structures in the lower parts of panels a & b are not described. The meaning of each of the colors should be explained, for example, in panel c there is yellow in the lower panel but not in the upper panel so it is difficult to connect what is being shown in each panel.*

   - **Response**
     Done

7. *Page 12, paragraph 3, first sentence: There is a typo "Our simulated p53-ASPP ensemble binding complexes unify the and crystallography- and NMR- modes" should be "Our simulated p53-ASPP ensemble binding complexes unify the crystallography and NMR binding modes."*

   - **Response**
     Thanks for pointing out, and we appreciate your careful reading and valuable suggestions.

8. *Page 12, paragraph 4, first sentence: "reversal" should be "reversible".*

   - **Response**
     Done

9. *Page 12, paragraph 4: "which states that dynamic IDP increases the searching radius to capture binding partner" should be "which states that dynamic IDPs increase the search radius to capture their binding partners"*

   - **Response**
     Done

10. *Page 12, paragraph 4, last sentence: "indicating more different samplings.." should be "indicating a difference in the binding interactions sampled."*

    - **Response**
      Done

11. *11. Page 12, paragraph 5, last sentence: "that from which" should be "from which".*

    - **Response**
      Done

0.2 Peer Reviewer #2:

1. *The authors state the respective binding free energies of ~7.57 kcal/mol and ~12.65 kcal/mol, are significantly different. There are no error estimates provided for the values (nor are any shown in the PMF plots), so it is impossible to tell whether this difference is indeed significant (larger than the estimated error). The PMF plot suggest so, but mentioning the estimated error in the text helps the reader.*

   - **Response**
     We thank the reviewer's comments on statistical rigorousness of the data presentation. The errors on the PMF curves were estimated by the built-in Monte Carlo bootstrapping algorithm of WHAM program by setting *num_MC_trials=100*. Briefly, it generated resamplings of the RC data points and calculated the corresponding PMF profile, and repeated this trials by user-designated times to obtain PMF errors.
     After obtaining the PMF curves (Fig. 4A), the binding free energy is defined as $\Delta G = PMF_{bound} - PMF_{unbound}$, where bound and unbound states were set at $RC = 1.5$ Å and $RC = 14.5$ Å, respectively. With error propagation, we obtained significantly

different ΔG values of -12.73 ± 0.12 kcal/mol and -7.57 ± 0.11 kcal/mol for ASPP2 and iASPP, respectively. We have added the error estimation details in the Method section **See tag R2Q1 on Page 12, Line 560**

2. *How have error estimations been calculated? That is not given in the methods section.*
   - **Response**
     Thanks for the question. Briefly, errors were estimated using the bootstrapping strategy. Besides the answer to question 1, we have added in the Method section about error estimation on Martini-related quantity (e.g., the inter-protein contact frequency, first contact formation time etc): we employed a bootstrapping procedure to obtain 10000 resamplings (sample size equals 50) from the original 50 simulation replicas, thereby to estimate the error. **See tag R1Q3 on Page 13, Line 591**
3. *Figure 4(B) needs a colour bar that explains the meaning of the blue, greenish to red areas.*
   - **Response**
     Thanks for the suggestion. We have added color bar to clarify that these different colors correspond to relative population densities.
4. *What does ns mean in Figure 4(D)?*
   - **Response**
     In the original Fig.4D, "ns" means "not significant". We have redrawn Fig. 4D
5. *What are the error estimates for encounter times (Fig. 5A)) ?*
   - **Response**
     Please refer to question 2.
6. *The PMFs shown in Fig S2 may have error estimates shown as shaded area, but this is unclear to see and not mentioned in the caption.*
   - **Response**
     Thanks for the question. We have redrawn all the error bars and explicitly mentioned these errors (how they were estimated, and their magnitudes) in the figure caption.

Abstract

ASPP2 and iASPP bind to p53 through their conserved ANK-SH3 domains to respectively promote and inhibit p53-dependent cell apoptosis. While crystallography has indicated that these two proteins employ distinct surfaces of their ANK-SH3 domains to bind to p53, solution NMR data has suggested similar surfaces. In this study, we employed multi-scale molecular dynamics (MD) combined with free energy calculations to reconcile the discrepancy in the binding modes. We demonstrated that the binding mode based solely on a single crystal structure does not enable iASPP's RT loop to engage with p53's C-terminal linker—a verified interaction. Instead, an ensemble of simulated iASPP-p53 complexes facilitates this interaction. We showed that the ensemble-average inter-protein contacting residues and NMR-detected interfacial residues ~~align well with~~ qualitatively overlap on ASPP proteins, and the ensemble-average binding free energies better match experimental Kd values compared to single crystallgarphy-determined binding mode. For iASPP, the sampled ensemble complexes can be grouped into two classes, resembling the binding modes determined by crystallography and solution NMR. We thus propose that crystal packing shifts the equilibrium of binding modes towards the crystallography-determined one. Lastly, we show that the ensemble binding complexes are sensitive to p53's intrinsically disordered regions (IDRs), attesting to experimental observations that these IDRs contribute to biological functions. Our results provide a dynamic and ensemble perspective for scrutinizing these important cancer-related protein-protein interactions (PPIs).

1 Introduction

The tumor suppressor protein p53 is a transcription factor that activates genes involved in apoptosis and cell-cycle arrest upon the detection of oncogenic stress.[1] p53 is a signaling hub that can be regulated by numerous proteins (involved in >1000 protein-protein interactions (PPIs)[2]). ~~Consequence~~ The main consequence of p53 ~~activation is majorly~~ is cell apoptosis, but it can also cause cell-cycle arrest, depending on specific p53 regulators.[3] ASPP2 and iASPP proteins are two p53 regulators that promote and inhibit p53-dependent apoptosis, respectively.[4] Given the critical role p53 plays in ~~tumor~~tumors, mechanical insights into the reversal regulatory effects of ASSP2 and iASPP on p53 are ~~much wanted yet are obscured by lacking of definite structural characterizations of the PPI.~~ of great importance for potential therapeutic developments. However, mechanical understanding is obscured, in part, due to controversial binding modes that have been reported on these PPIs.

So far, structural characterizations have primarily focused on interactions within the folded portions, specifically between p53's DNA-binding domain (DBD) and the ankyrin repeats and an SH3 domain (ANK-SH3) of iASPP/ASPP2[5,6] (Fig. 1A). The ANK-SH3 domains of ASPP2 and iASPP are highly conserved, sharing approximately 70% sequence identity and nearly identical crystal structures (Cα RMSD $\sim$ 1.24 Å).[6,7] Despite this high degree of conservation, crystallography has revealed that when binding to p53's DBD, ASPP2 and iASPP employ entirely different surfaces on their ANK-SH3 domains: ASPP2 primarily utilizes the SH3 domain to bind p53, while iASPP employs a flat surface formed by the parallel ank-repeat helices to interact with p53 (Fig. 1B). This significant difference in binding modes has been reported to contribute to ASPP2 and iASPP's opposing regulatory effects on p53. ASPP2 directly displaces DNA from p53's DBD through a competitive binding mechanism,[8,9] whereas iASPP binds to p53, triggering a molecular switch in the p53 L1 loop, interfering with p53's DNA binding.[6] While the general mechanism by which ASPP proteins alter p53's binding capacity to various genes, thus influencing cellular life-or-death processes,[6,10,8] has been established, this mechanism is challenged by conflicting reports on the binding modes of iASPP-p53 interactions. Ahn et al.'s solution NMR data suggests that iASPP uses a similar binding surface on its ANK-SH3 domain as ASPP2 to interact with p53's DBD[4] (Fig. 1B), in stark contrast to the crystallography-determined iASPP-p53 complex (PDB 6RZ3).

Discrepancies between crystallography and NMR structures are frequently encountered, especially when determining biologically relevant PPIs.[11,12,13,14] Both methods have their own advantages and shortcomings. While the structures themselves are accurate, the protein-protein complexes determined by crystallography may not be biologically relevant due to crystal packing effects.[11] This is especially true for weak PPIs, as it has been reported that there is a greater than 50% probability that crystallography-determined complexes are not biologically relevant if the dissociation constant (K_D) is greater than 100 μM.[11] In contrast, solution NMR captures protein motion in an aqueous environment and is therefore more functionally related. However, it suffers from inaccuracies in chemical shift assignments and cannot directly provide atomistic structures; instead, it serves as constraints to derive structures that satisfy the observed chemical shifts.[12] Solution NMR generally reflects ensemble-average properties, as demonstrated by Pochapsky *et al*, who used solution NMR to capture multiple p450 protein conformers that are all important to the protein's function.[15] Meanwhile, the binding mode determined by crystallography is more likely to be a subset of the ensemble of binding complexes, as demonstrated by Zuo ~~et al.~~*et al*, who used computational methods to show that multiple poses, including the X-ray one, are all consistent with experimental affinities.[16] For our system of interest, whether the observed discrepancies between crystallography and NMR in determining the binding mode for iASPP-p53 are complementary or mutually exclusive is unknown and warrants further exploration.

Reconciling the discrepancy in binding modes can provide valuable insights into the roles of intrinsically disordered regions (IDRs) in regulating the ASPP-p53 PPI. NMR studies have suggested that p53's IDRs play a role in fine-tuning the PPI. Both ASPP and p53 encompass significant portions of their protein sequences predicted as IDRs. Ahn et al. demonstrated that ASPP2 primarily binds to p53's DNA-binding domain (DBD), while iASPP predominantly interacts with a linker region located C-terminal to the DBD domain.[4] This highlights the influence of p53's IDRs in distinguishing between bindings to the highly conserved ANK-SH3 domains of ASPP2 and iASPP. Furthermore, in the case of ASPP2-p53 binding, solution NMR studies conducted by Tidow et al.[8] revealed that, in addition to the interface residues reported in PDB 1YCS, p53 residues outside the interface (such as loop 1 and the C-terminal helix) were implicated in binding ASPP2. Clarifying the relevance of these NMR-based observations to ASPP-p53 functions is expected to be possible if the aforementioned binding mode discrepancy is resolved.

In this study, we performed extensive multi-scale molecular dynamics (MD) simulations to investigate if p53's DBD domain and ASPP2/iASPP ANK-SH3 domain could bind in multiple binding modes, and whether the controversial crystallography- and NMR- determined binding modes can be reconciled under this ensemble binding framework. We also simulated the bindings

of different p53 constructs that contain extra IDRs to ASPP, to explore how p53's IDRs fine-tuning the ASPP-specific PPIs. We anticipate that our structural characterizations could facilitate thearpeutic developments targeting these important PPIs.

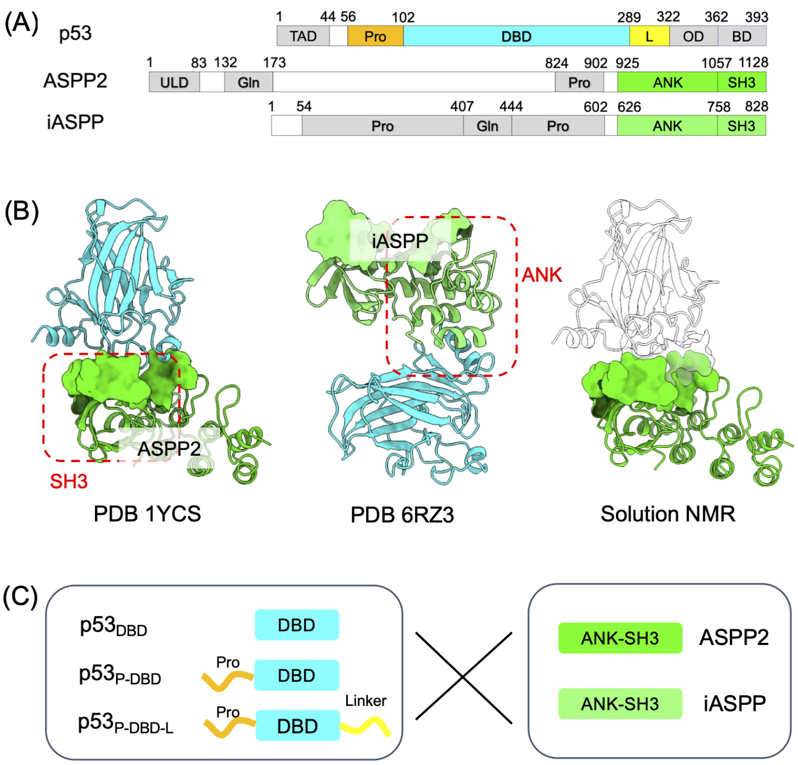

Figure 1: (A) Domain organizations of p53 and ASPP proteins. p53's DBD and ASPP's ANK-SH3 are folded domains whereas the remaining sequences are intrinsically disordered. B) Crystal structures of ASPP2-p53 complex (PDB 1YCS[5]) and iASPP-p53 complex (PDB 6RZ3[6]). Solution NMR studies[4] also suggest a iASPP-p53 binding mode that is different from PDB 6RZ3. (C) In this study, we combined all-atom and coarse-grained (CG) MDs plus free energy calculations to explore the binding mechanisms between three p53 constructs ($p53_{DBD}$ $p53_{P\text{-}DBD}$ and $p53_{P\text{-}DBD\text{-}L}$) and the ANK-SH3 domains of ASPP2/iASPP.

2 Materials and methods

2.1 Conventional molecular dynamics (MD) simulations

Simulations were based on the crystal structures of $p53_{DBD}$ in complex with ASPP2 and iASPP's ANK-SH3 domains (PDB IDs are 1YCS[5] and 6RZ3,[6] respectively). The input files for MD were prepared by the TLEAP program from Amber20 package.[17] The system was solvated into a 12Å margin OPC waterbox with 0.15 M KCl ionic strength, and the Amber ff19SB force field[18] was used for protein. The system was first subject to 50000 steps energy minimization with the first 200 steps using the steepest descent algorithm and the remaining steps using the conjugate gradient algorithms. The minimized system was then heated to 300 K via a two-step procedure: 0 to 100 K in NVT ensemble over 0.1 ns followed by 100 to 300 K in NPT ensemble over 0.5 ns. During heating, harmonic constraints with a force constant of 5 kcal/mol/$\AA^2$ were introduced onto protein backbone atoms. After heating, a 1 ns equilibrium simulation was performed in the NPT ensemble at 300 K with reduced force constant of 1 kmol/mol/$\AA^2$. Three independent 1 μs long production runs (NPT ensemble, 300 K) were initiated from equilibrated system. During the simulation, all of the length of bonds involving hydrogen were restrained by the SHAKE algorithm,[19] and the temperature was controlled by Langevin thermostat[20], **R1Qm4** and the Particle-Mesh Ewald

(PME) method[21] was used to handle long-range electrostatic interactions. The time step was 4 fs after hydrogen mass repartitioning[22] and the nonbonded cutoff was 12.0 Å.

Notably, the bound zinc ion in p53 crystal structures was considered in our all-atom MD simulations. This Zn^{2+} is vital to p53's structure stability and function,[23,24] and our own test in Fig. S2 shows that removing the Zn^{2+} alters the dynamics of $p53_{DBD}$ and changes the conformation of motifs at the PPI interface with ASPPs. However, since protein force fields generally notoriously handle polar contacts[25] , to maintain the coordination of Zn^{2+} to p53's His83-CYS80,142,146 motif, we introduced harmonic constraints between Zn^{2+} ion and the coordinating atoms with a force constant of 300 kcal/mol/Å^2 to retain Zn^{2+} binding. The parameters of Zn^{2+}, as well as the KCl monovalent ions, were adapted from the Li-Merz dataset.[26]

Besides the $p53_{DBD}$, we built two additional p53 constructs that have the IDR regions flanking p53's DBD domain added: The $p53_{P\text{-}DBD}$ that has the N-terminal Pro-domain (residues 56-102) added, and $p53_{P\text{-}DBD\text{-}L}$ that has the additional C terminal linker (residues 289-322) added. The initial structures of these IDRs were built from sequences using the TLEAP program, and were sampled from 500 ns conventional MD at 300 K in explicit solvent model. **R1Q1a** While this 500 ns long conventional MD is generally insufficient to thoroughly explore the conformational space of these IDRs, it provides relatively stable structures (e.g., local energy minima conformations) for the subsequent simulations to start with. We showed in Fig. S3 that the RMSD and radius of gyration (RoG) of the IDRs are approaching steady over the 500 ns conventional MDs, suggesting stable conformations are achieved for these IDRs. The representative structure of the most-populated cluster was selected, and was then joined to $p53_{DBD}$ using Pymol. Simulations of these p53 constructs' interactions with ASPPs were started from the above mentioned PDB structures and followed the same MD protocol.

2.2 Martini CGMD to simulate the binding process between p53 and ASPP

The Martini3.0 coarse-grained (CG) model[27] was used to simulate the protein-protein binding. +Coarse graining of p53 and ASPP all-atom structures into Martini beads were performed using the VERMOUTH program,[28] and the DSSP program[29,30] was used to detect protein secondary structure information. The bound Zn^{2+} ion in p53 was omitted for coarse ~~graning~~graining, thanks to the elastic network strategy that can maintain protein tertiary structure. **R1Q1b** Notably, for p53 constructs, we excluded the IDRs from the elastic network constraints to retain the dynamic nature of the IDRs. We showed in Fig. S3 that this strategy indeed allowed p53's IDRs to sample various conformations prior to binding to ASPP2/iASPP. The elastic bond force constants was set as 500 kJ/mol/nm^2 and the lower- and upper- elastic bond cutoffs were 5 and 9 Å, respectively. Since long range electrostatic interactions between proteins become centrysymmetric at $\sim$40 Å,[31] the ASPP ANK-SH3 domain was randomly placed >=40 Å away from p53 construct to minimize bias. The system was solvated in a 150×150×150 Å cubic box with 0.15 M ionic strength using standard Martini water and NaCl models.[32] The system was first energy minimized 2000 steps using the steepest descent algorithm and was then heated to 300 K in the NPT ensemble over 1 ns, with constraints on the protein. The equilibrated system was subject to a 4 μs production MD in NPT ensemble at 300 K using a 20 fs time step. The temperature control was achieved with the velocity rescale (V-rescale) and pressure was controlled with the Parrinello-Rahman barostat. For each system, 50 replicas of 4 μs CGMD runs were performed via Gromacs2020.4[33] to investigate the binding between p53 constructs and ASPP ANK-SH3 domains.

2.3 Umbrella samplings to obtain the ~~disassociation~~ dissociation potential of mean force (PMF)

Umbrella samplings were performed to estimate the potential of mean force (PMF) along the ~~disassociation~~ dissociation pathway of the p53-ASPP complexes, starting from both crystal complex structures and Martini CGMD sampled complexes. Martini complexes were backmapped into all-atom structures using the BACKWARD.PY[34] script, and were subject to 100 ns explicit solvent MD with Zn^{2+} added to p53 to further optimize the complex following the abovementioned MD protocol. The reaction coordinate (RC) was defined as distance between centers of mass (COM) of p53 and ASPP and was binned into 0.5 Å-wide windows. Samplings in each window was enforced by harmonic constraints with 10 kcal/mol/Å^2 force constant. Starting structures used for each window are the last trajectory frames from previous windows. For each window, a 10 ns production MD was performed in NPT ensemble at 300 K. **R2Q1**The PMF was estimated by the WHAM program[35]~~.~~ , and the errors on the PMF curves were estimated by the built-in

Monte Carlo bootstrapping algorithm of WHAM program by setting *num_MC_trials=100*. Briefly, it generated resamplings of the RC data points and calculated the corresponding PMF profile, and repeated this trials by user-designated times to estimate PMF errors. All MD simulations performed for this work are summarized in Table S1.

2.4 Sketch-map projections to obtain 2D mode

The sketch-map dimensionality reduction method[36,37] was employed to project the Martini CGMD sampled ASPP-p53 complexes onto 2D plane. Each trajectory frame was encoded as a high-dimensional space vector, $\vec{X_D} = (D_1, D_2, ..D_N)$, where $D_1, D_2...D_N$ are the minimum distances between the backbone beads (Martini BB type) of p53 and ASPP. Beads were chosen from the secondary structure elements (helices and sheets) from p53 and ASPP because inter-protein distances between these rigid structures have low fluctuations compared with loops, and can thus accurately encode the binding configurations.[38] We set N as 21: D_1 to D_{10} represents the minimum distance of the 10 residues of p53 to 11 residues of ASPP.Namely, for residue i, we first calculated its distance to the selected ASPP residues and assign the smallest value to D_i. And D_{11} to D_{21} are the reverse minimum distances, namely, the minimum distance between ASPP residues to p53. Projection follows the principle that points close in the high-dimensional space should be close in the 2D space,[36] by minimizing the following stress function:[36]

$$\chi^2 = \frac{\sum_{j\neq i} w_i w_j [F(R_{ij}) - f(r_{ij})]}{\sum_{j\neq i} w_i w_j} \tag{1}$$

where w_i, w_j are weights of points, R_{ij}, r_{ij} are distances between points in the high- and low-dimensional spaces, respectively. F, f are sigmoid functions that map distances between 0 and 1. In our calculations, points weight and sigmoid functions were taken as the default values from the sketch-map tutorial.

2.5 Analyses

Analyses were conducted using the CPPTRAJ program,[39] MDTraj,[40] and Matplotlib libraries. RMSD, RMSF and distance calculations were conducted using CPPTRAJ. 3D space distributions of p53 around ASPP were estimated using the *grid* command from CPPTRAJ. We selected a 0.5 Å spacing to build 3D grids centered on the average COM position of ANK-SH3 domain after aligning the MD trajectories on this domain, and then binned p53 densities into grids, and normalized to standard water density 1.0 g/cm^3. Projections of MD trajectories onto 2D plane were done using the MDTraj library which aligns trajectories and extracts the Cartesian coordinate of protein atoms. **R1Q3** Inter-protein contact data was calculated using the *nativecontacts* command from CPPTRAJ with default distance cutoff 7 Å. The encounter time was defined as the time when the first inter-protein contact forms during the Martini CGMD. The means and standard error of means of these quantities were estimated via a bootstrapping procedure to obtain 10000 resamplings (sample size equals 50) from the original 50 simulation replicas. Structure were rendered using UCSF ChimeraX,[41,42] and VMD.[43] Scripts supporting this work are available at https://github.com/bsu233/bslab/tree/main/2023-p53ASPP.

3 Results

3.1 MD simulations starting from crystallography binding modes fail to capture the experiment-verified inter-protein interactions

Both NMR studies[4] and peptide screening assays[44] have confirmed that p53 utilizes its intrinsically disordered linker (located C-terminal to the DBD domain) to bind to iASPP's RT loop. A designed peptide mimicking p53's linker has been shown to competitively displace p53 from binding to iASPP, thereby blocking iASPP's regulation of p53 in vitro.[44] We conducted MD simulations to investigate whether p53's linker can interact with iASPP's RT loop in the crystal structure-resolved binding modes, aiming to justify the relevance of these modes as the biological unit. We initiated our simulations from both PDB 1YCS and 6RZ3, assuming that p53 can bind to iASPP via these two modes. The samplings presented in Fig. 2 clearly demonstrate that, starting from both crystal structure-resolved binding modes, p53's linker is unable to reach iASPP's RT loop. The discrepancies observed between the MD samplings and experimental findings strongly suggest that iASPP and p53 likely engage in alternative binding modes.

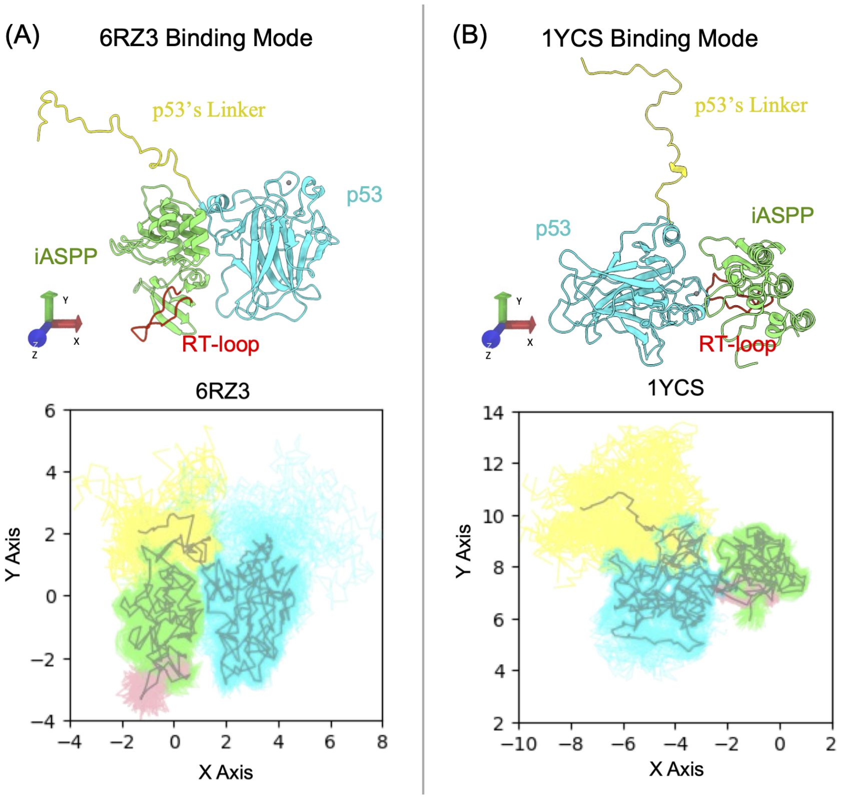

Figure 2: Examining the interactions between p53's C-terminal linker (yellow) and iASPP's RT loop (red), using 3×1 μs all-atom MDs. (A) Starting from PDB 6RZ3. (B) Starting from PDB 1YCS. For each case, MD trajectories were concatenated and were aligned on p53's DBD against the shown respective reference structure. The X and Y Cartesian coordinates of the protein backbone atoms were used for the projection. ASPP and p53 are colored green and cyan, respectively. The gray lines depict the average structures of protein backbone.

3.2 Ensemble-average interprotein contacts agree with solution NMR revealed PPI interfaces

To explore potential alternative binding modes between p53 and ASPP, we conducted unbiased coarse-grained (CG) molecular dynamics simulations (MD) to simulate the protein-protein binding processes. We opted for the Martini 3.0 CGMD model[27] due to its consideration of side-chain packing, which has been shown to have a critical impact on protein-protein interactions.[45] We first simulated the binding between $p53_{\text{P-DBD}}$ and the ANK-SH3 domains of ASPPs. Solution

NMR studies conducted by Ahn *et al*[4] reported chemical shift changes in ASPPs residues ~~caused by the binding of~~ induced by p53 binding, allowing us to map out the interfacial residues on the ANK-SH3 domains. We therefore calculated the inter-protein ~~contacts~~ contact frequency based on the cumulative Martini CG trajectories and compared the per-residue ~~contacts~~ contact frequency of ANK-SH3 domain residues with the NMR data~~to validate our Martini CG results~~, as these two quantities are closely related in annotating PPI surface residues.

~~As illustrated~~ **R1Q3a** As shown in Fig. 3~~A-B, for both ASPP2~~ A, overall the Martini CG captures the major interfacial residues as detected by NMR. To quantify the overlap of CG and NMR, we calculated the RMSE (root mean square error) as $RMSE = \sqrt{\sum_i (CG_i - NMR_i)^2/N}$, where NMR_i and ~~iASPP, the per-residue contacts of the ANK-SH3 domain qualitatively agree with the NMR chemical shift data~~ CG_i are NMR chemical shift and Martini normalized contact frequency (average value after bootstrapping) for all ASPP residues. We reported RMSE = 0.069 and 0.059 for iASPP and ASPP2, respectively. Our interpretations are simulated results and NMR data have moderate deviation for both ASPPs, with iASSP being slightly more severe. However, for iASPP, our Martini CG captures almost all of the crystal structure (PDB 6RZ3) determined interfacial residues, as the red "*"-marked residues are encompassed by Martini CG data (Fig. 3A). Lastly, although CG and NMR data do show moderate deviations, after mapping them onto ASPP proteins, the overall interfacial regions detected by NMR are qualitatively captured by Martini CG simulations (Fig. 3B). In the case of ASPP2, the contact data suggests that the borders between ANK repeats, particularly the ANK3-ANK4 border, exhibit significant contacts with p53. Furthermore, the SH3 domain is largely involved in contacting p53. These contact patterns are consistent with the NMR chemical shift data. For iASPP, both Martini CGMD and NMR data indicate that the SH3 domain is at the protein-protein interface. Additionally, Martini CGMD reports that ANK4 is involved in interacting with p53 (~~Figure 3~~Fig. 3B), partially resembling the inter-protein interaction observed in the p53$_{\mathrm{DBD}}$-iASPP complex crystal structure (PDB 6RZ3). This suggests that the ensemble of p53-iASPP structures sampled using Martini CGMD may contain the binding mode observed in the crystal structure (which will be discussed in detail later). Overall, we observed that the binding complexes obtained from Martini CGMD qualitatively capture the protein-protein interface reflected in the solution NMR data.

Having validated the Martini CGMD methodology for characterizing PPIs, we proceeded to simulate the binding between p53$_{\mathrm{P\text{-}DBD\text{-}L}}$ and iASPP to investigate whether p53's C-terminal linker interacts with iASPP's RT loop, as experimentally demonstrated.[4,44] We analyzed the atom densities of p53$_{\mathrm{P\text{-}DBD\text{-}L}}$ around iASPP, calculated from the accumulated Martini CGMD trajectories (Fig. 3C), and observed that p53 is positioned around iASPP's RT loop. Projections of these samplings onto a 2D plane further elucidated the specific interactions. These data provide compelling evidence that the simulated binding complexes accurately capture the interactions between p53's linker and iASPP's RT loop, in agreement with experimental findings.

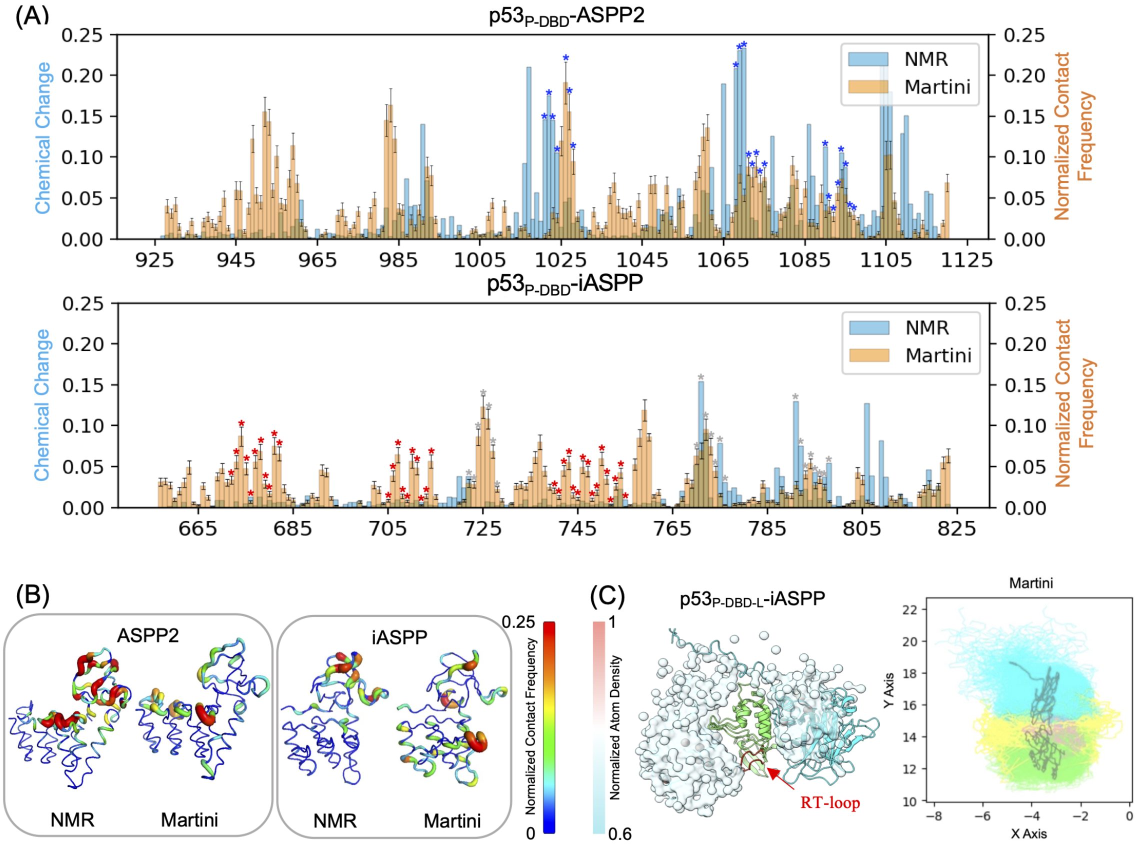

Figure 3: (A) Comparison of NMR-detected residue chemical shift changes and Martini CGMD sampled residue contact frequency on the ANK-SH3 domains of ASPP. NMR is normalized to highest chemical shift change value. Martini contact frequency was calculated by first summing all contacts involving each ASPP residue and divided by the total number of frames, followed by normalization to $C_{max} = 0.0002$. ASPP residues that are involved in interacting with p53 as determined by crystallography were marked by differently-colored asterisks: blue from PDB 1YCS (ASPP2-p53$_{\text{DBD}}$), red from PDB 6RZ3 (iASPP-p53$_{\text{DBD}}$), and gray from the NMR-suggested 1YCS-like binding mode for iASPP. (B) Mapping the data shown in panel A onto the ANK-SH3 domain structures. (C) Martini CGMD sampled p53$_{\text{P-DBD-L}}$-iASPP complexes. The normalized atoms densities of p53 in the 3D space around ASPP, and the projections of samplings onto the XY plane were shown to the left and right, respectively. The p53's C-terminal linker (yellow) is interacting with iASPP's RT loop (red).

3.3 Ensemble-average binding free energy agrees with experimental affinities

We have demonstrated that the ensemble binding complexes qualitatively recapitulate the results of solution NMR and peptide screening assays. Now, we aim to strengthen these outcomes quantitatively. $p53_{DBD}$ binds to the ANK-SH3 domains of ASPP2 and iASPP with comparable affinities (K_Ds are 26.4 ± 1.7 nM and 23.3 ± 1.6 nM, respectively[7]). Consequently, we calculated the potential of mean force (PMF) for dissociation and compared it against these experimental K_Ds to validate the binding modes.

~~Firstly, when starting from the crystal-structure-captured single binding mode, iASPP exhibits significantly weaker affinity than ASPP2 in binding , with binding free energies of −7.57~~ Based on the dissociation PMF curves (Fig. 4A), we defined the binding free energy as $\Delta G = PMF_{bound} - PMF_{unbound}$, where bound and unbound states were set at $RC = 1.5$ Å and $RC = 14.5$ Å, respectively. With error propagation, we obtained significantly different ΔG values of -12.73 ± 0.12 kcal/mol and ~~−12.65~~ -7.57 ± 0.11 kcal/mol ~~, respectively (A). This~~ for ASPP2 and iASPP, respectively. This suggested that iASPP exhibits significantly weaker affinity than ASPP2 in binding $p53_{DBD}$. This discrepancy contradicts the comparable experimental K_D values. To investigate if starting from multiple binding poses can mitigate this discrepancy, we conducted simulations of the binding process between $p53_{DBD}$ and the ANK-SH3 domains using unbiased Martini CGMD and analyzed the binding modes from various perspectives. **R1Q4** We projected the Martini CG trajectories onto a 2D plane using the inter-protein distance and RMSD to iASPP/ASPP2-$p53_{DBD}$ cocrystal structures as axes. These two axes are helpful to assess overall distributions of sampled complexes. Interestingly, for both ASPPs, Martini can capture crystal-like binding modes with RMSD as low as 3.2 Å (Fig. 4B), ~~even though these modes are not the predominant binding modes.~~ although the major simulated bound complexes lie in the 25<distance< 50 Å, and 12 <RMSD< 22 Åregions. To further examine whether the recaptured binding modes are non-specific coincidences resulting from extensive simulation replicas or ASPP-specific binding modes arising from unique protein-protein interactions, we plotted the overall atom density distributions of $p53_{DBD}$ around ASPP (Fig. 4C). This clearly shows that $p53_{DBD}$ exhibits different distribution patterns when bound to ASPP2 and iASPP: Martini-sampled $p53_{DBD}$ conformers are continuously distributed around ASPP2, in contrast to the "bi-sided" distributions around iASPP (Fig. 4C). This suggests that the sampled binding modes are ASPP isoform-dependent. Notably, the "bi-sided" distributions of $p53_{P\text{-}DBD\text{-}L}$ around iASPP correspond to the major binding modes observed in crystal structures 6RZ3 and 1YCS, implying that iASPP may bind to $p53_{DBD}$ in two predominant modes.

Lastly, we selected multiple representative Martini CGMD-sampled $p53_{DBD}$-ASPP complexes and performed umbrella samplings to obtain the dissociation PMFs. We employed a rigorous selection procedure to maximize the representativeness of the selected binding complexes (see Section Sect. S2). As shown in Fig. 4D, ASPP2 exhibits different distributions of binding free energies compared to iASPP: ASPP2 ~~includes several cases with weak binding, which are compensated by tight binding ones~~binders are more evenly distributed in the weak-strong range, while iASPP cases ~~are more concentrated around the experimental K_Ds. However, the average values of~~ tend to concentrate onto the two ends of affinity spectrum. **R1Q7** Intriguingly, the binding affinities of the two iASPP poses based on crystal structures also follow this pattern, as they show a ΔG difference of 14.6 kcal/mol (Fig. S4), resembling the weak and strong binders we selected for iASSP from Martini CGMD-sampled complexes. **R1Q6** We performed the t-test between CG simulation-generated poses and crystal structures for iASPP2 and ASPP2, and t-test between various calculated binding free energies ~~show no statistically significant difference (P~~ and experimental affinities (Fig. 4D). For ASPP2, for which the reported crystallography binding mode is consistent with solution NMR data, the crystal structure based ΔG and CG-simulated poses based ΔG are not significantly different ($p =$ ~~0.995, t-test) . Calculated binding~~ 0.284). For iASPP, while the reported crystal structure (PDB 6RZ3) also does not show significant $DeltaG$ difference with that from Martini simulations ($p = 0.438$), its another NMR-suggested binding mode (PDB 1YCS) is significantly different from the Martini ΔG ($p = 0.0008$). Importantly, for both ASPP2 and iASPP, ΔGs calculated from crystal structures are significantly different from the experimentally-measured binding affinities. However, the ΔGs based on Martini simulation poses are not statistically different from experimental values, with p=0.623 and 0.781 for ASPP2, and iAPP, respectively. These data suggest that calculated binding free energies starting from crystal structures do not align with the experimental observations that iASPP and ASPP2 have comparable binding affinities toward $p53_{DBD}$. Instead, the ensemble-average energies based on Martini CGMD-sampled multiple binding poses align with the experimental observations.

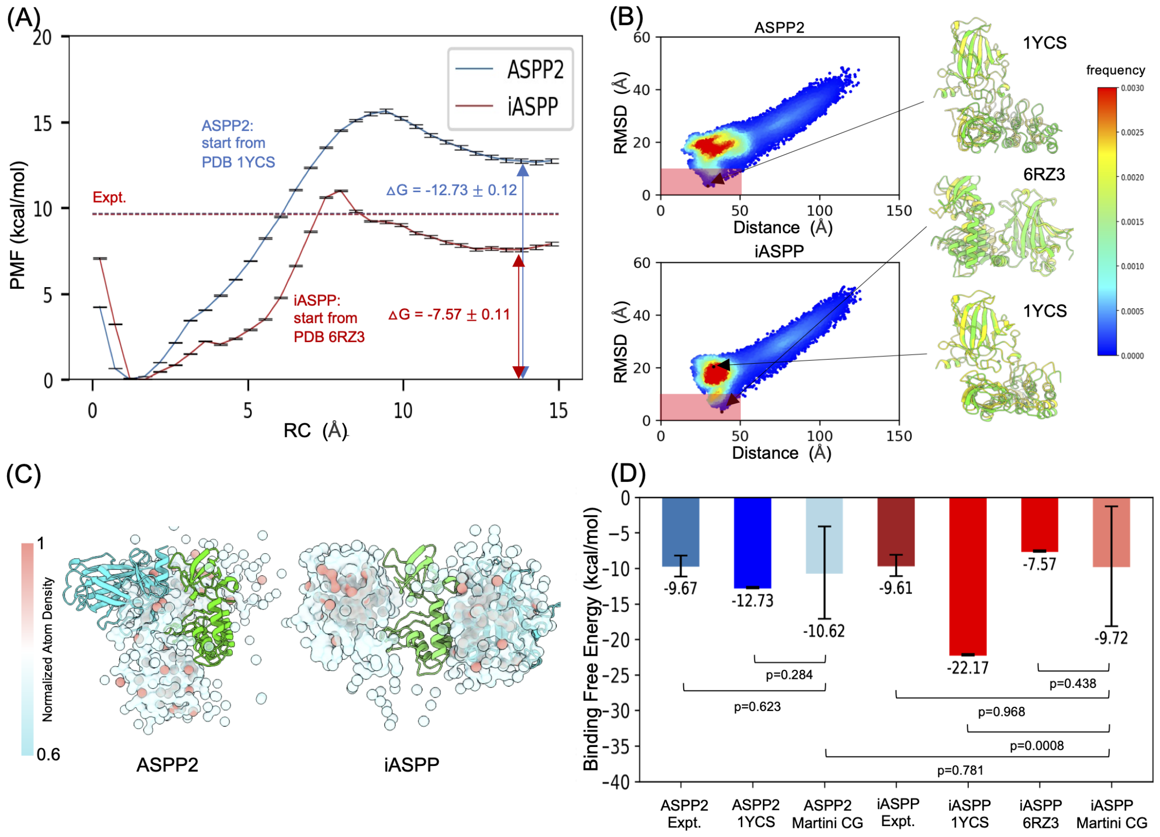

Figure 4: (A) PMF curves of the dissociation process between $p53_{DBD}$ and ASPPs, starting from the crystal-structure binding modes. The PMF errors were estimated by the Monte Carlo bootstrapping procedure of the WHAM program. PMF-derived binding free energy (ΔG) were calculated by defining the bound state at $RC = 1.5$ Å and the unbound state at $RC = 14.5$ Å. The experimentally measured ΔGs were shown as red dashed lines. (B) Martini CGMD simulated binding between $p53_{DBD}$ and ASPPs. The trajectories are projected onto the distance (COM of $p53_{DBD}$ to COM of ANK-SH3 domain), and the RMSD (with respect to crystal structures) axes. The color scale means frequency of binding. The red shaded areas highlight the binding poses that are close to crystal structures, and those resemble exactly to crystal structures were superimposed onto crystal structures. (C) Overall atom density distribution of $p53_{DBD}$ (cyan) around ASPP (green) in the 3D space. (D) Comparison of experimentally measured ΔG, calculated ΔGs based on crystal structures, and ΔGs calculated from multiple representative Martini complexes (detailed PMF curves are shown in Fig. S7). t-tests were performed to assess the statistical significance.

3.4 p53's IDRs accelerate the binding rates and further differentiates p53's bindings toward ASPP2 and iASPP

We further investigated the impact of the intrinsically disordered regions (IDRs) in the p53 protein, which constitute approximately 48% of the entire protein (Fig. S1), on the PPIs based on our Martini CG trajectories. We calculated the time of formation of first inter-protein contact to assess how IDRs in our system affect protien-protein binding rates. Comparison between p53$_{\text{DBD}}$ and p53$_{\text{P-DBD-L}}$ shows that addition of IDRs to p53$_{\text{DBD}}$ significantly reduces the mean first passage time for inter-protein contact formation, from approximately ~~130~~ 542 ns to approximately ~~90~~ 370 ns (Fig. 5A). **Q1Q8a** To verify if the greatly shortened "first contact formation time" for IDR-containing constructs may be just due to larger size of the p53 constructs that allow ASPP to more easily interact, rather than is the dynamic nature of IDRs that accelerates the binding, we recalculated the first contact formation time between p53 DBD domain and ASPP even if p53 IDRs are present.We observed that counting only p53's DBD domain interacting with ASPP still shows comparable first contact formation time as counting the whole p53's constructs. This indicates an enhancement in the association rates facilitated by the presence of IDRs. Moreover, we performed one additional set of Martini CGMD simulations in which p53 IDRs were rigidified via constraints. This was to validate if the dynamic nature of the IDRs affect the binding rates. We observed that treating IDRs as rigid motifs reverse the binding accelerating phenomena, resulting in a much slower binding for the p53$_{\text{P-DBD-L}}$. These data suggest that at local structural level, the p53's DBD, regardless the presence of IDRs, is the predominant domain to first interact with ASPPs. More importantly, the shortened first contact formation time, thereby the accelerated binding kinetics of the p53$_{\text{P-DBD}}$ and p53$_{\text{P-DBD-L}}$ relative to p53$_{\text{DBD}}$ are caused by the dynamics of IDRs.

R1Q11 Interestingly, while the addition of IDRs to p53 does not ~~significantly alter the kinetics of binding to~~ differentiate the binding patterns towards ASPP2 and iASPP kinetically, it does influence the specific inter-protein contacts in the simulated complexes. We calculated the time-dependent and p53-domain-specific inter-protein contacts (Fig. 5B). ~~These results clearly demonstrate that the presence of~~ **R1Q9** Firstly, it shows that p53 ~~'s IDRs leads to distinct interactions~~DBD's interaction with both ASPPs are attenuated when p53 IDRs are present. Secondly, the degrees of DBD domain de-involvement are different for ASPP2 and iASPP: When binding to ASPP2, p53's DBD domain maintains considerable contacts, whereas for binding to iASPP, the interaction relies less on the DBD domain and is primarily mediated by p53's linker (Fig. 5B). These results clearly demonstrate that the presence of p53's IDRs leads to distinct interactions.

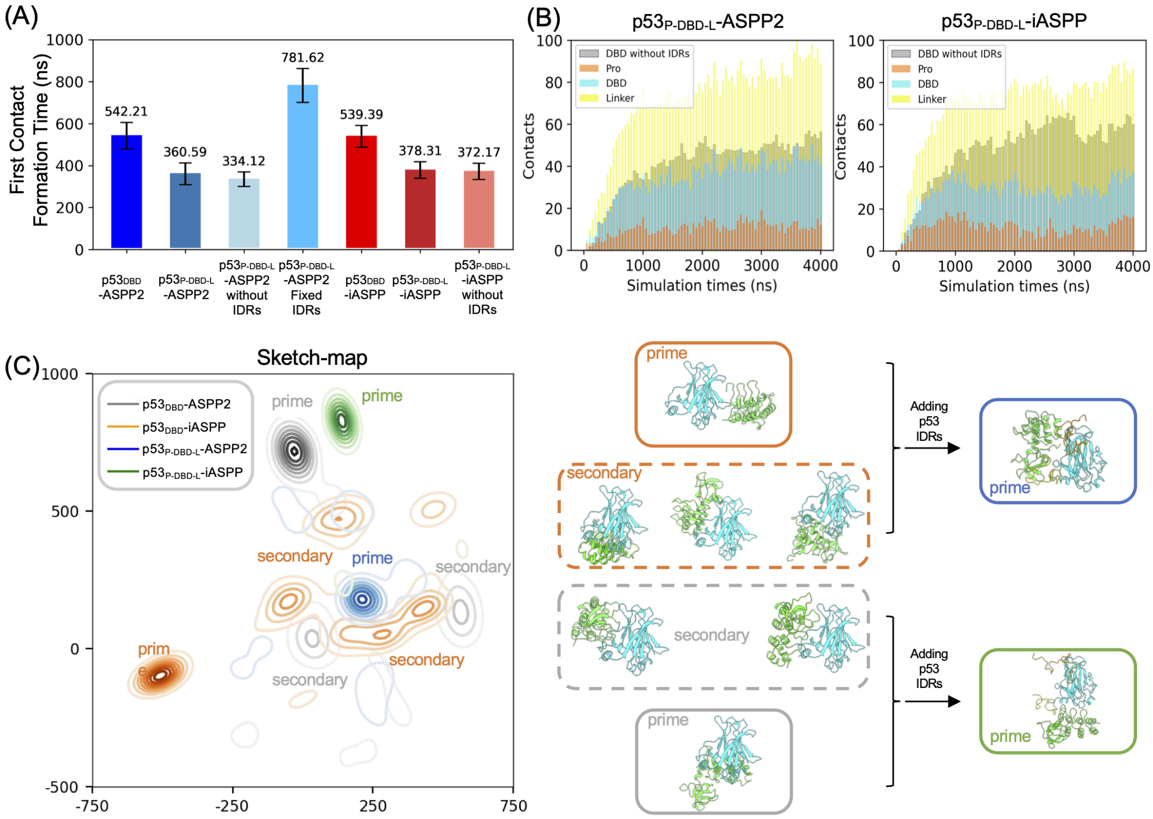

Figure 5: (A) First contact formation times of ASPPs binding to p53$_{\text{DBD}}$ and p53$_{\text{P-DBD-L}}$. For p53$_{\text{P-DBD-L}}$, besides the normal definition of first contact as "any ASPP residue to any p53 residue", we also counted the first contact defined as "any ASPP residue to residues only from p53 DBD domain". For ASPP2-p53$_{\text{P-DBD-L}}$ binding, an additional set of 50×4 μs Martini CG was performed in which p53's IDRs were rigidified by constraints (the "Fixed IDRs" bar, using the normal definition of first contact). (B) Average time-dependent number of contacts between ASPP and different p53 domains of p53$_{\text{P-DBD-L}}$ were calculated from 50×4 μs Martini CG simulations. The contacts based on p53$_{\text{DBD}}$-ASPP simulations were also shown as gray bars. (C) Projections of Martini CGMD sampled ASPP-p53 complexes onto 2D plane using the sketch-map dimentionality reduction method. The "prime" and "secondary" binding modes refer to the most highly-sampled states, and the less sampled but still have considerable population states (if present), respectively. Representative complexes were shown right to the sketch-map projection.

Furthermore, the presence of p53's linker serves to differentiate the binding patterns between iASPP and ASPP2 when interacting with p53. To illustrate this, we employed the sketch-map dimensionality reduction method to project the simulated ASPP-p53 complexes onto a 2D plane. The choice of sketch-map enables precise projection of the relative configurations of ASPP-p53 complexes in 3D space onto the 2D plane. Consequently, points that are close in the 2D plane represent similar ASPP-p53 binding configurations, allowing for straightforward comparison of the simulated ASPP-p53 complexes. As shown in Fig. 5C, in the absence of IDRs, the binding of $p53_{DBD}$ to iASPP2 and ASPP2 exhibits moderate overlap, despite differing major binding poses (i.e., high-density areas differ for iASPP2 and ASPP2). Notably, the addition of IDRs to $p53_{DBD}$ leads to a dramatic change in the distribution patterns for both iASPP and ASPP2 on the 2D projection plane. This results in more concentrated and distinct binding patterns for these two ASPPs. **R1Q10** To gain structural insights into how the p53's IDRs fine-tune the interactions, we reported representative complexes that correspond to the high-density areas in sketch-map plots (Fig. 5). Consistent with our interpretations on sketch-map, we show that, in the absence of IDRs, although the prime binding modes of ASPP2 and iASPP towards $p53_{DBD}$ are different, their secondary binding modes have similar complexes. Adding IDRs diminishes the overlap and leaves the utterly unlike prime binding modes of ASPP2 and iASPP. These projection data strongly suggest that the inclusion of p53's IDRs further differentiates p53's binding interactions with ASPP2 and iASPP.

4 Discussion

Two lines of evidences suggested that the iASPP-p53 binding mode may be different from that resolved in PDB 6RZ3, and this alternative mode is highly similar to the ASPP2-p53 binding mode resolved in PDB 1YCS. Firstly, the isolated iASPP ANK-SH3 crystal structure (PDB 2VGE[7]) was found to use a surface patch, which resembles the ASPP2's binding patch towards p53, to interact with neighbouring unit. Secondly, solution NMR data shows that the p53-induced residue chemical shift perturbations were mapped onto the similar surfaces for ASPP2 and iASPP ANK-SH3 domains.[4] The ongoing debate over the binding mode presents challenges to achieving a comprehensive understanding of ASPP-p53 regulations.

Discrepancies between NMR and crystallography-determined modes are not uncommon in the literature, not only for individual proteins but also for protein-protein interactions (PPIs). While NMR and crystallography-derived protein structures align closely in most cases,[12] certain instances can exhibit substantial structural deviations, with backbone RMSD reaching up to 6 Å.[13] This divergence can be attributed to the distinct environments in which the structures are determined, namely, aqueous solution versus crystal lattice. Regarding PPIs, assigning solution NMR-determined PPIs as biologically relevant protein binding modes is generally less contentious. However, the same cannot be said for crystallography-determined PPIs due to the influence of crystal packing. Crystal packing effects can introduce free energy advantages that compete with native PPI formations.[11,46,47] Consequently, the relevance of crystallography-determined PPIs to biological functional complexes is affinity-dependent.[46] Strong PPIs (e.g., K_D <100 μM[46]) are more likely to retain their native binding modes within crystal lattices. **R1Q13** In our specific case, the binding affinities ~~between~~ of p53 ~~'s DBD and~~ constructs, especially for the p53$_{\text{P-DBD}}$, towards ASPP's ANK-SH3 domains ~~are around the μM range as measured by different methods, which is in proximity to the~~ can approach the 100 μM threshold~~.~~[4,8]. This brings uncertainty in assuring the crystal structure captured ASPP-p53 complexes as biological-relevant assembly, especially for the iASPP-p53 PPI.

Our simulated p53-ASPP ensemble binding complexes unify the ~~and~~ crystallography- and NMR- modes. Firstly, we demonstrated that the ensemble of binding complexes better aligns with experimental observations, supported by two key arguments: 1) the ensemble binding modes satisfy the experimental-confirmed binding patterns while the single crystal structure does not. 2) Calculated binding free energies, using representative complexes from the ensemble, closely match the experimental K_D values. More importantly, the simulated complexes contain the exact crystal-structure binding modes, and for iASPP, the simulated complexes exhibit "bi-sided" distributions ~~-~~of p53 around the ANK-SH3 domain, which can be treated as two generalized binding modes representing the NMR-suggested and crystallography-captured binding mode, respectively. Therefore, p53$_{\text{DBD}}$ binds to iASSP's ANK-SH3 domain in two configurations in solution, and crystal packing shifts the binding equilibrium to the one captured in PDB 6RZ3. This potentially elucidates why iASPP utilizes similar binding modes as ASPP2 towards p53, as suggested by solution NMR, while exhibiting a different binding mode in the crystal structure. As a result, our simulated ASPP-p53 complexes are complementary to the NMR and crystallography characterizations of the p53-ASPP complexes.

IDRs are not merely flexible but have envolved to exert unique functions such as allowing rapid and ~~reversal~~ reversible PPI and acting as recognition motifs.[48,49] Our simulation data suggest that p53's IDRs play multiple roles in fine-tuning the PPI. IDPs are well-known to accelerate protein binding rates through the "fly-casting" mechanism[50] which states that dynamic ~~IDP increases the searching~~ IDPs increase the search radius to capture ~~binding partner~~their binding partners. This was reflected in our simulations as we show that adding the N-terminal Pro-domain and C-terminal linker to p53$_{\text{DBD}}$ greatly accelerates the binding to both ASPP and iASSP. **R1Q8b** We additionally confirmed that the rate-accelerating effect originates from the dynamic nature of p53 IDRs by showing that rigidifying the IDRs through constraints totally reverse the rate-accelerating effects. **R1Q14c** While the rate-accelerating effect brought by p53's IDRs are similar between ASPP2 and iASPP, the simulated protein-protein binding complexes ~~(e.g., the encounter complexes)~~ are differently impacted by p53's IDRs. To visualize the difference, we elected to use the sketch-map dimensionality reduction method to project the ASPP-p53 complex conformation onto the 2D plane. We show that adding IDRs to p53$_{\text{DBD}}$ significantly reduced the overlap between ASPP2 and iASPP, indicating ~~more different samplings. .~~ a difference in the binding interactions sampled.

R1Q15 One angle to explain the functional consequence of differently-sampled ASPP-p53 ~~encounter~~ complexes is through the ~~concept of~~ "~~effective concentration~~refolding of encounter complexes" ~~.~~ subprocess of protein-protein binding [51,52,45]. **R1Q12** Although we implicitly treated the Martini CGMD sampled complex as "fully bound complexes" in previous sections, a close look at the time-dependent binding process reveals that these simulated complexes were experiencing different degrees of refolding (Fig. 6). While in some cases the ASPP and p53 did

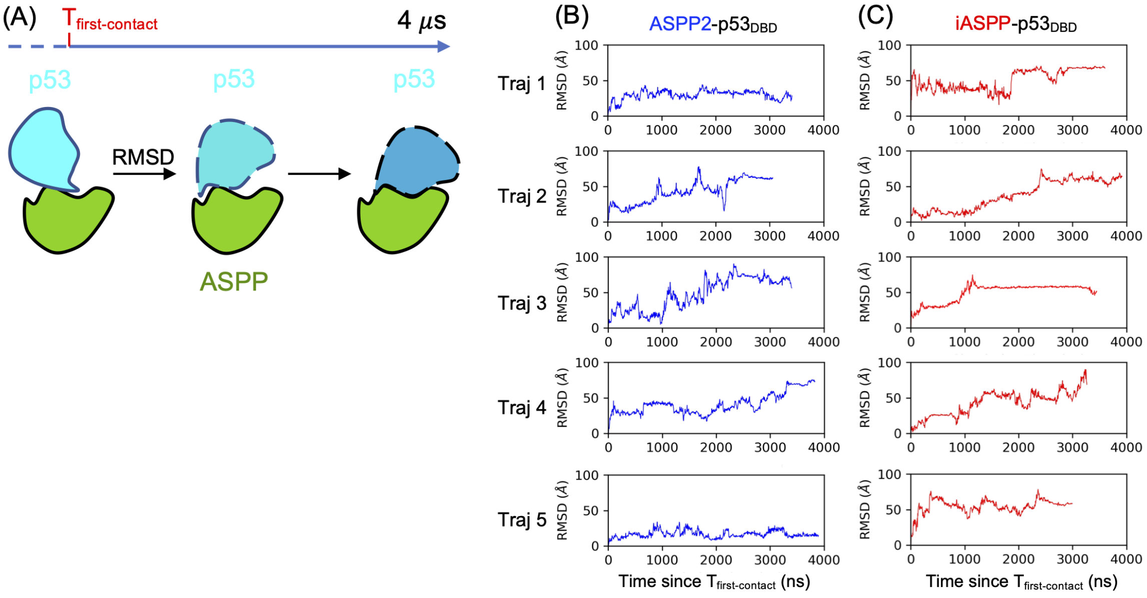

Figure 6: Assessing the refolding of Martini CGMD sampled ASPP-p53 complexes. A) Refolding is gauged by a RMSD metric, namely, starting from the moment when ASPP and p53 first contact, the subsequent complexes' RMSDs with respect to the first contact complex were calculated. (B-C) Five Martini CGMD trajectories were randomly selected to assess the refolding for ASPP2 and iASPP in the binding of $p53_{DBD}$ respectively.

not refold (e.g, bind and stick together), in most simulation trajectories, these two protein had, and would likely to continue, the refoldings. We speculate that over much longer time-scale, most of our Martini CGMD sampled complexes would continue to refold and converge to fewer binding modes that are more biologically-relevant. From this perspective, the Martini CGMD complexes can be loosely treated as "encounter complex" that would continue their journey to the native modes. ~~Protein-protein binding process can be divided into two sub-processes : 1) Diffusion~~ In this context, our Martini CGMD amounts to complete the diffusion of protein partners to form ~~loosely bound complexes (encounter complexes). 2) Rearrangement of encounter complex~~ the encounter complexes [45], which await the rearrangements to reach native PPI state, such as rolling on another protein's surface.[53] ~~While step one is driven by diffusion, step two (refolding)~~ Since we have proved that p53's IDRs can fine-tune the protein-interaction patterns, then it is likely that adding IDRs can change the portions of simulated complexes that can successfully reach the destined states. This is because the refolding process is conditional, and occurs only on subsets of the encounter ~~complex~~ complexes that satisfy certain criteria such as shape ~~complementary~~ complementarity .[54] In this regard, adding IDRs to p53 increase the size of the candidate pool ~~(effective concentration) that~~ from which more complexes can refold to the native PPI state.

However, the mechanism by which ensemble binding of PPIs exerts its biological functions remains elusive, and addressing this question requires further structural characterizations encompassing the entire ASPP and p53 proteins. Our argument is supported by several studies demonstrating that motifs distant from the folded domains contribute to their biological function. For example, peptide screening assays have shown that ASPP2's Pro-domain can autoinhibit the ANK-SH3 domain, competing with p53's binding.[48] Additionally, an ASPP2 isoform with the N-terminal region deleted inhibits p53-dependent apoptosis, exhibiting a completely different regulatory effect on p53 compared to full-length ASPP2.[55] Even for ASPP1 and ASPP2 proteins, both of which promote p53-dependent apoptosis and have nearly identical sequences and folds in their ANK-SH3 domains, there is a ∼10-fold difference in their affinities for p53, with K_D=5 μM for ASPP2 and K_D=0.5 μM for ASPP1, respectively. However, ASPP2 can displace the DNA promoter from the p53-DNA promoter complex, while ASPP1 cannot.[9] This contradiction, where ASPP2 has weaker affinity with p53 but can still displace the DNA promoter, implies that other parts of ASPP are involved in *in vivo* regulation. Therefore, a comprehensive understanding of the contrasting regulatory effects of ASPP2 and iASPP on p53 requires structural and biological investigations at the whole protein level in PPIs.

5 Conclusions

In this study, we performed multi-scale MDs and free energy calculations to characterize the PPI of two proteins that of great therapeutic potentials, the p53 and its regulator(s), iASPP/ASPP2. Our work focus on reconciling the discrepancy between NMR- and crystallography- determined binding modes for the iASPP-p53 PPI. We used unbiased Martini3.0 CGMD to simulated the protein-protein PPI, and achieved the ASPP2 and iASPP-specific ensemble binding modes that can unify the reported binding mode discrepancy. We first validated the ensemble binding modes by showing that the ensemble-average inter-protein contacts are well aligned with solution NMR-detected chemical shift perturbations caused by p53. Furthermore, the ensemble-average PPI binding free energies agree with experimental Kds. Notably, we observed that the crystallography-determined binding modes were recapitulated within the ensemble-binding modes, indicating that the crystal packing environment may influence the equilibrium of binding modes toward the configuration captured by crystallography. Although linking these ensemble binding modes to actual biological functions poses a challenge, we provided evidence that the ensemble binding modes are highly sensitive to p53's intrinsically disordered regions (IDRs). The inclusion of IDRs accentuated the differences in binding modes exhibited by p53 towards ASPP2 and iASPP. This sensitivity underscores the biological significance of ensemble bindings and is consistent with observations indicating that the entire proteins, rather than just their folded domains, contribute to biological functions.

6 Acknowledgements

Research reported in this work was supported by the Harbin Medical University high-level introduction of talent research start-up fund (No.310212000109, 31011210004) and the National Science Foundation (No. 22002096).

References

[1] Yardena Samuels-Lev et al. "ASPP proteins specifically stimulate the apoptotic function of p53". In: *Molecular cell* 8.4 (2001), pp. 781–794.

[2] Yaw Sing Tan, Yasmina Mhoumadi, and Chandra S Verma. "Roles of computational modelling in understanding p53 structure, biology, and its therapeutic targeting". In: *Journal of molecular cell biology* 11.4 (2019), pp. 306–316.

[3] Yeyun Zhou et al. "Flexible Tethering of ASPP Proteins Facilitates PP-1c Catalysis". In: *Structure* 27 (10 2019), 1485–1496.e4. ISSN: 0969-2126.

[4] Jinwoo Ahn et al. "Insight into the structural basis of pro- and antiapoptotic p53 modulation by ASPP proteins". In: *Journal of Biological Chemistry* 284 (20 2009), pp. 13812–13822. ISSN: 00219258.

[5] Svetlana Gorina and Nikola P Pavletich. "Structure of the p53 tumor suppressor bound to the ankyrin and SH3 domains of 53BP2". In: *Science* 274.5289 (1996), pp. 1001–1005.

[6] Shuo Chen et al. "iASPP mediates p53 selectivity through a modular mechanism fine-tuning DNA recognition". In: *Proceedings of the National Academy of Sciences* 116.35 (2019), pp. 17470–17479.

[7] Ross Alexander Robinson et al. "Biochemical and Structural Studies of ASPP Proteins Reveal Differential Binding to p53, p63, and p73". In: *Structure* 16 (2 2008), pp. 259–268. ISSN: 09692126.

[8] Henning Tidow et al. "Effects of Oncogenic Mutations and DNA Response Elements on the Binding of p53 to p53-binding Protein 2 (53BP2) *". In: *Journal of Biological Chemistry* 281 (43 2006), pp. 32526–32533. ISSN: 0021-9258.

[9] Seema Patel et al. "Molecular interactions of ASPP1 and ASPP2 with the p53 protein family and the apoptotic promoters PUMA and Bax". In: *Nucleic Acids Research* 36 (16 2008), pp. 5139–5151. ISSN: 0305-1048.

[10] Yardena Samuels-Lev et al. "ASPP proteins specifically stimulate the apoptotic function of p53". In: *Molecular cell* 8.4 (2001), pp. 781–794.

[11] Evgeny Krissinel. "Crystal contacts as nature's docking solutions". In: *Journal of Computational Chemistry* 31.1 (2010), pp. 133–143.

[12] G Wagner, S G Hyberts, and T F Havel. "NMR Structure Determination in Solution: A Critique and Comparison with X-Ray Crystallography". In: *Annual Review of Biophysics and Biomolecular Structure* 21.1 (1992). PMID: 1525468, pp. 167–198.

[13] John K. Everett et al. "A community resource of experimental data for NMR / X-ray crystal structure pairs". In: *Protein Science* 25.1 (2016), pp. 30–45.

[14] Steve P Meisburger, David A Case, and Nozomi Ando. "Robust total X-ray scattering workflow to study correlated motion of proteins in crystals". In: *Nature Communications* 14.1 (2023), p. 1228.

[15] Thomas C. Pochapsky and Susan Sondej Pochapsky. "What Your Crystal Structure Will Not Tell You about Enzyme Function". In: *Accounts of Chemical Research* 52.5 (2019). PMID: 31034199, pp. 1409–1418.

[16] Ke Zuo et al. "Predictions of the Poses and Affinity of a Ligand over the Entire Surface of a NEET Protein: The Case of Human MitoNEET". In: *Journal of Chemical Information and Modeling* 63.2 (2023). PMID: 36623826, pp. 643–654.

[17] DA Case et al. "AMBER2020, university of California, San Fransisco". In: *J. Amer. Chem. Soc* 142 (2020), pp. 3823–3835.

[18] Chuan Tian et al. "ff19SB: Amino-acid-specific protein backbone parameters trained against quantum mechanics energy surfaces in solution". In: *Journal of chemical theory and computation* 16.1 (2019), pp. 528–552.

[19] Jean-Paul Ryckaert, Giovanni Ciccotti, and Herman JC Berendsen. "Numerical integration of the cartesian equations of motion of a system with constraints: molecular dynamics of n-alkanes". In: *Journal of computational physics* 23.3 (1977), pp. 327–341.

[20] Ruslan L Davidchack, Richard Handel, and MV Tretyakov. "Langevin thermostat for rigid body dynamics". In: *The Journal of chemical physics* 130.23 (2009).

[21] Tom Darden, Darrin York, and Lee Pedersen. "Particle mesh Ewald: An N log (N) method for Ewald sums in large systems". In: *The Journal of chemical physics* 98.12 (1993), pp. 10089–10092.

[22] Chad W Hopkins et al. "Long-time-step molecular dynamics through hydrogen mass repartitioning". In: *Journal of chemical theory and computation* 11.4 (2015), pp. 1864–1874.

[23] Jianxin Duan and Lennart Nilsson. "Effect of Zn2+ on DNA recognition and stability of the p53 DNA-binding domain". In: *Biochemistry* 45.24 (2006), pp. 7483–7492.

[24] James S Butler and Stewart N Loh. "Structure, function, and aggregation of the zinc-free form of the p53 DNA binding domain". In: *Biochemistry* 42.8 (2003), pp. 2396–2403.

[25] Robin Rainwater et al. "Role of cysteine residues in regulation of p53 function". In: *Molecular and cellular biology* 15.7 (1995), pp. 3892–3903.

[26] Pengfei Li et al. "Rational design of particle mesh Ewald compatible Lennard-Jones parameters for+ 2 metal cations in explicit solvent". In: *Journal of chemical theory and computation* 9.6 (2013), pp. 2733–2748.

[27] Paulo CT Souza et al. "Martini 3: a general purpose force field for coarse-grained molecular dynamics". In: *Nature methods* 18.4 (2021), pp. 382–388.

[28] Peter C Kroon et al. "Martinize2 and Vermouth: Unified Framework for Topology Generation". In: *arXiv preprint arXiv:2212.01191* (2022).

[29] Wouter G Touw et al. "A series of PDB-related databanks for everyday needs". In: *Nucleic acids research* 43.D1 (2015), pp. D364–D368.

[30] Wolfgang Kabsch and Christian Sander. "Dictionary of protein secondary structure: pattern recognition of hydrogen-bonded and geometrical features". In: *Biopolymers: Original Research on Biomolecules* 22.12 (1983), pp. 2577–2637.

[31] Bin Sun et al. "Electrostatic control of calcineurin's intrinsically-disordered regulatory domain binding to calmodulin". In: *Biochimica et Biophysica Acta (BBA)-General Subjects* 1862.12 (2018), pp. 2651–2659.

[32] Semen O Yesylevskyy et al. "Polarizable water model for the coarse-grained MARTINI force field". In: *PLoS computational biology* 6.6 (2010), e1000810.

[33] Lindahl et al. *GROMACS 2020.4 Manual*. Version 2020.4. 2020.

[34] Tsjerk A Wassenaar et al. "Going backward: a flexible geometric approach to reverse transformation from coarse grained to atomistic models". In: *Journal of chemical theory and computation* 10.2 (2014), pp. 676–690.

[35] Alan Grossfield. "WHAM: the weighted histogram analysis method, version". In: ().

[36] Michele Ceriotti, Gareth A Tribello, and Michele Parrinello. "Simplifying the representation of complex free-energy landscapes using sketch-map". In: *Proceedings of the National Academy of Sciences* 108.32 (2011), pp. 13023–13028.

[37] Gareth A Tribello, Michele Ceriotti, and Michele Parrinello. "Using sketch-map coordinates to analyze and bias molecular dynamics simulations". In: *Proceedings of the National Academy of Sciences* 109.14 (2012), pp. 5196–5201.

[38] Andrej Berg and Christine Peter. "Simulating and analysing configurational landscapes of protein–protein contact formation". In: *Interface focus* 9.3 (2019), p. 20180062.

[39] Daniel R Roe and Thomas E Cheatham III. "PTRAJ and CPPTRAJ: software for processing and analysis of molecular dynamics trajectory data". In: *Journal of chemical theory and computation* 9.7 (2013), pp. 3084–3095.

[40] Robert T McGibbon et al. "MDTraj: a modern open library for the analysis of molecular dynamics trajectories". In: *Biophysical journal* 109.8 (2015), pp. 1528–1532.

[41] Thomas D Goddard et al. "UCSF ChimeraX: Meeting modern challenges in visualization and analysis". In: *Protein Science* 27.1 (2018), pp. 14–25.

[42] Eric F Pettersen et al. "UCSF ChimeraX: Structure visualization for researchers, educators, and developers". In: *Protein Science* 30.1 (2021), pp. 70–82.

[43] William Humphrey, Andrew Dalke, and Klaus Schulten. "VMD: visual molecular dynamics". In: *Journal of molecular graphics* 14.1 (1996), pp. 33–38.

[44] Guy Mayer et al. "Targeting an Interaction Between Two Disordered Domains by Using a Designed Peptide". In: *Chemistry–A European Journal* 26.45 (2020), pp. 10240–10249.

[45] Bin Sun and Peter M. Kekenes-Huskey. "Assessing the Role of Calmodulin's Linker Flexibility in Target Binding". In: *International Journal of Molecular Sciences* 22.9 (2021). ISSN: 1422-0067.

[46] Mitchell R. OĆonnell, Roland Gamsjaeger, and Joel P. Mackay. “The structural analysis of protein–protein interactions by NMR spectroscopy”. In: *PROTEOMICS* 9.23 (2009), pp. 5224–5232.

[47] Fei Tan and Jin Xu. “Validation of the solution structure of dimerization domain of PRC1”. In: *Plos one* 17.8 (2022), e0270572.

[48] Shahar Rotem-Bamberger, Chen Katz, and Assaf Friedler. “Regulation of ASPP2 interaction with p53 core domain by an intramolecular autoinhibitory mechanism”. In: *PLoS One* 8.3 (2013), e58470.

[49] Bin Sun and Peter M. Kekenes-Huskey. “Myofilament-associated proteins with intrinsic disorder (MAPIDs) and their resolution by computational modeling”. In: *Quarterly Reviews of Biophysics* 56 (2023), e2.

[50] Benjamin A Shoemaker, John J Portman, and Peter G Wolynes. “Speeding molecular recognition by using the folding funnel: the fly-casting mechanism”. In: *Proceedings of the National Academy of Sciences* 97.16 (2000), pp. 8868–8873.

[51] G. Schreiber, G. Haran, and H.-X. Zhou. “Fundamental Aspects of ProteinProtein Association Kinetics”. In: *Chemical Reviews* 109.3 (2009). PMID: 19196002, pp. 839–860.

[52] Dima Kozakov et al. “Encounter complexes and dimensionality reduction in protein–protein association”. In: *eLife* 3 (2014). Ed. by Benoit Roux, e01370. ISSN: 2050-084X.

[53] Ali S. Saglam and Lillian T. Chong. “Protein–protein binding pathways and calculations of rate constants using fully-continuous, explicit-solvent simulations”. In: *Chem. Sci.* 10 (8 2019), pp. 2360–2372.

[54] Raik Grünberg, Johan Leckner, and Michael Nilges. “Complementarity of Structure Ensembles in Protein-Protein Binding”. In: *Structure* 12.12 (2004), pp. 2125–2136. ISSN: 0969-2126.

[55] Kathryn Van Hook et al. “DeltaN-ASPP2, a novel isoform of the ASPP2 tumor suppressor, promotes cellular survival”. In: *Biochemical and Biophysical Research Communications* 482.4 (2017), pp. 1271–1277. ISSN: 0006-291X.

[56] Ethan Garner et al. “Predicting binding regions within disordered proteins”. In: *Genome informatics* 10 (1999), pp. 41–50.

[57] Andreas C Joerger, Hwee Ching Ang, and Alan R Fersht. “Structural basis for understanding oncogenic p53 mutations and designing rescue drugs”. In: *Proceedings of the National Academy of Sciences* 103.41 (2006), pp. 15056–15061.

Supplementary Information (SI):

Reconciling ASPP-p53 Binding Mode Discrepancies through an Ensemble Binding Framework that Bridges Crystallography and NMR Data

Te Liu[1], Sichao Huang[1], Qian Zhang[1], Yu Xia[1], Manjie Zhang[1*] and Bin Sun[1*],

[1] Research Center for Pharmacoinformatics, College of Pharmacy, Harbin Medical University, Harbin 150081, China

[*] Corresponding author: zhangmj454@nenu.edu.cn binsun@hrbmu.edu.cn

Table S1: Molecular dynamics simulations performed in this study.

Simulation Types	Cases	Starting Structures	Simulation Length
conventional all-atom MD			
	$p53_{DBD}$ (w/o Zn^{2+})	PDB 1YCS	500 ns
	$p53_{DBD}$ (w/ Zn^{2+})	PDB 1YCS	500 ns
	$p53_{DBD}$-ASPP2 (w/ Zn^{2+})	PDB 1YCS	1 μs $\times$ 3
	$p53_{DBD}$-iASPP (w/ Zn^{2+})	PDB 6RZ3	1 μs $\times$ 3
	$p53_{P\text{-}DBD\text{-}L}$-iASPP (w/ Zn^{2+})	PDB 1YCS + modelled p53 IDRs	1 μs $\times$ 3
	$p53_{P\text{-}DBD\text{-}L}$-iASPP (w/ Zn^{2+})	PDB 6RZ3 + modelled p53 IDRs	1 μs $\times$ 3
Martini CGMD			
	$p53_{DBD}$-ASPP2	PDB 1YCS	4 μs $\times$ 50
	$p53_{DBD}$-iASPP	PDB 6RZ3	4 μs $\times$ 50
	$p53_{P\text{-}DBD}$-ASPP2	PDB 1YCS + modelled Pro-domain	4 μs $\times$ 50
	$p53_{P\text{-}DBD}$-iASPP	PDB 6RZ3 + modelled Pro-domain	4 μs $\times$ 50
	$p53_{P\text{-}DBD\text{-}L}$-ASPP2	PDB 1YCS + modelled Pro-domain and linker	4 μs $\times$ 50
	$p53_{P\text{-}DBD\text{-}L}$-ASPP2	PDB 1YCS + Rigidified IDRs	4 μs $\times$ 50
	$p53_{P\text{-}DBD\text{-}L}$-iASPP	PDB 6RZ3 + modelled Pro-domain and linker	4 μs $\times$ 50
all-atom umbrella samplings			
	$p53_{DBD}$-ASPP2 (w/o Zn^{2+})	PDB 1YCS	10 ns/win $\times$ ~30 win
	$p53_{DBD}$-ASPP2 (w Zn^{2+})	PDB 1YCS	10 ns/win $\times$ ~30 win
	$p53_{DBD}$-iASPP (w/o Zn^{2+})	PDB 6RZ3	10 ns/win $\times$ ~30 win
	$p53_{DBD}$-iASPP (w/ Zn^{2+})	PDB 6RZ3	10 ns/win $\times$ ~30 win
	$p53_{DBD}$-ASPP2 (w Zn^{2+})	Martini CGMD sampled complexes	10 ns/win $\times$ ~50 win $\times$ 20
	$p53_{DBD}$-iASPP (w/ Zn^{2+})	Martini CGMD sampled complexes	10 ns/win $\times$ ~50 win $\times$ 20

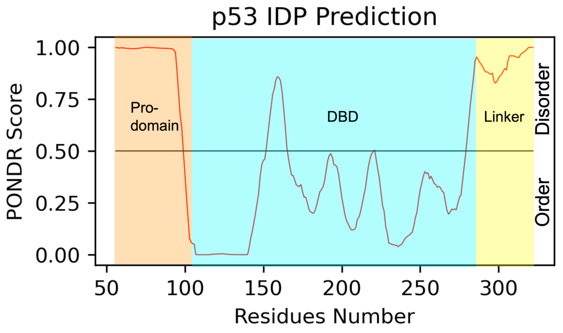

Figure S1: The intrinsically disordered domains of p53 as predicted by the online-website PONDR [56]. The Pro-domain and linker-domain flanking $p53_{DBD}$ are disordered.

S1 Zn^{2+} plays a critical role in stabilizing $p53_{DBD}$ and in affecting the binding free energy between $p53_{DBD}$ and ANK-SH3 domain of ASPP proteins

A Zn^{2+} cation was resolved in $p53_{DBD}$ crystal structure (PDB 1YCS) to coordinate with p53's His83-CYS80,142,146 motif. To investigate the importance of Zn^{2+}, we performed MD simulations on $p53_{DBD}$ with and without the Zn^{2+}, respectively. We found that removing Zn^{2+} leads to significant conformational change of $p53_{DBD}$ and alters the dynamics of the domain, as compared to the w/ Zn^{2+} case (Fig. S2A-B). Specifically, the L3 loop of $p53_{DBD}$ is distorted after removing the Zn^{2+}. This loop is at the PPI interface to bind ASPP2 (PDB 1YCS) [48], and has been shown to hub most cancer-causing mutations [57]. Therefore, Zn^{2+} role of stabilizing this loop needs to be considered. We also investigate the role of Zn^{2+} in affecting the potential of mean force (PMF) along the $p53_{DBD}$-ANK-SH3 dissociation pathway. As shown in Fig. S2C-D, presence of Zn^{2+} can significantly change the PMF curves for both ASPP2 and iASPP.

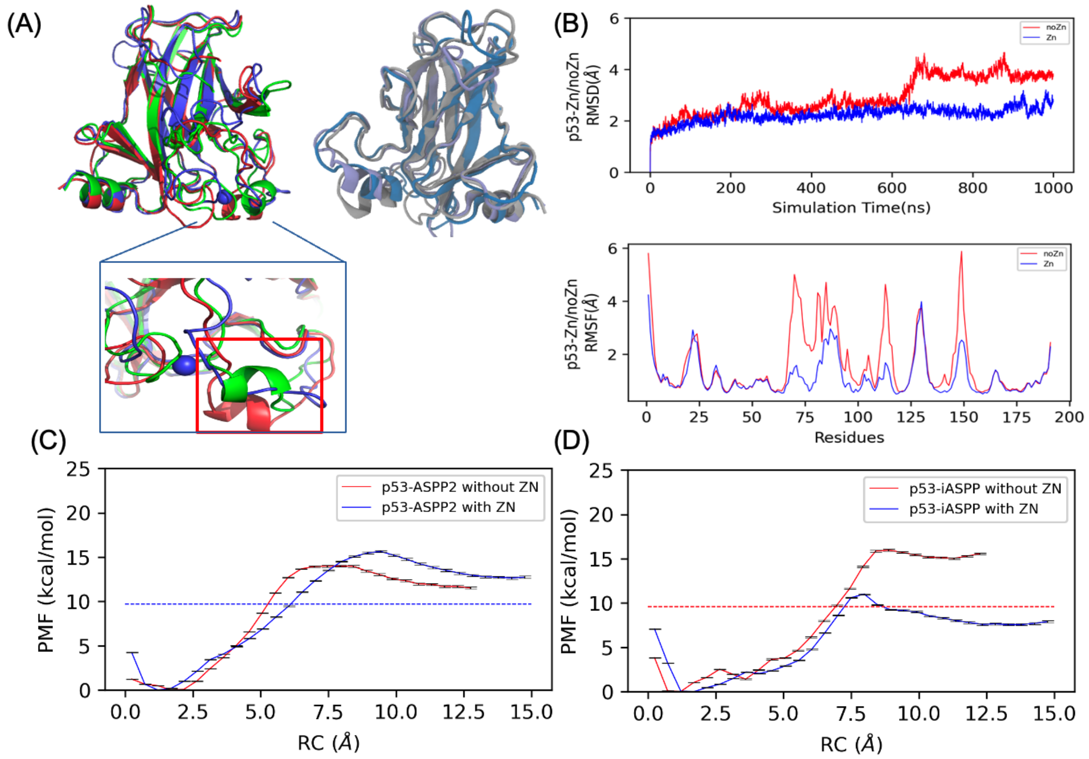

Figure S2: (A) Superimposing MD-sampled $p53_{DBD}$ conformations, w/ Zn^{2+} (blue) and w/o Zn^{2+} (red), on crystal structure (green). (B) RMSD and RMSF of $p53_{DBD}$ from MD simulations w/ and w/o Zn^{2+}. (C-D) PMF of $p53_{DBD}$ disassociation from ASPP2 and iASPP w/ and w/o Zn^{2+}. Errors bars were drawn in black, and were estimated by the built-in bootstrap error analysis of the WHAM program ($num\_MC\_trials = 100$). The relative small errors (~0.1 kcal/mol) reflect that our 10 ns samplings per window in the umbrella sampling is sufficient.

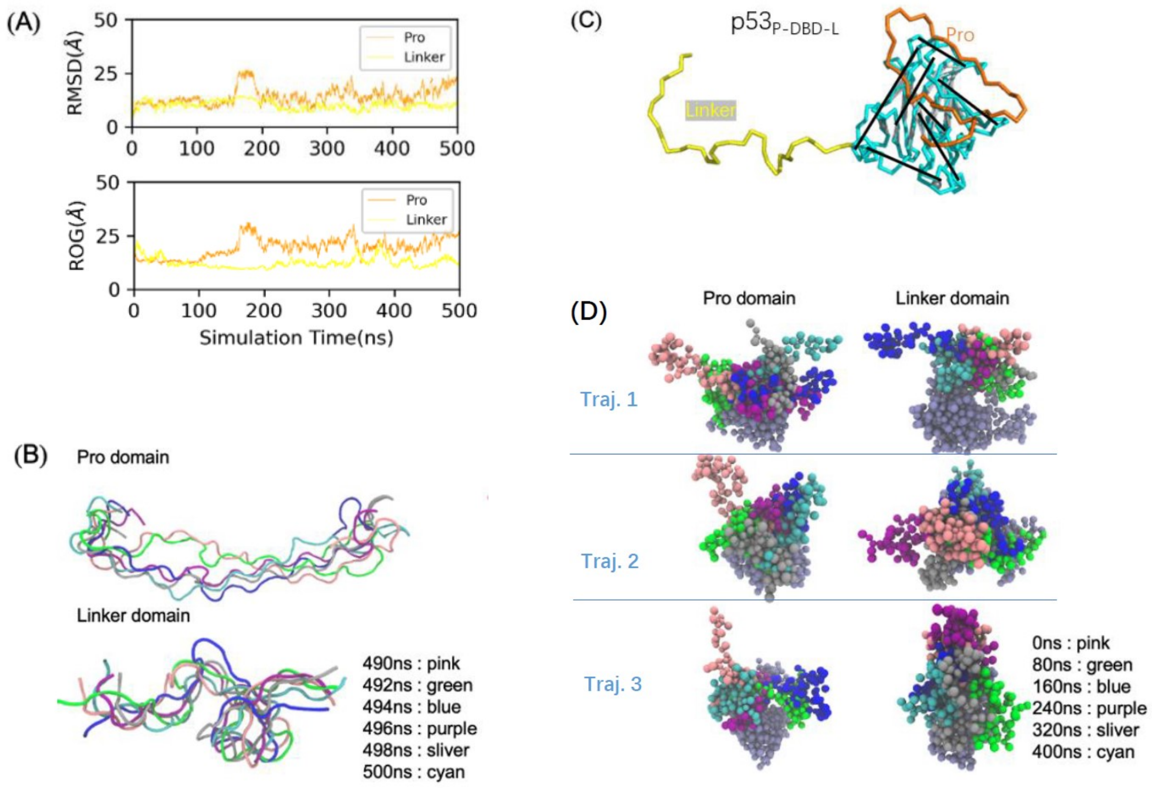

Figure S3: (A) RMSD and RoG (radius of gyration) of isolated p53 IDRs (Pro domain and linker domain) in 500 ns conventional all-atom MD. (B) Structures extracted from the last 10 ns trajectory of 500 ns MD at every 2 ns. (C) Schematic diagram showing the elastic networks that constrain only the DBD domain of $p53_{\text{P-DBD-L}}$ in the Martini CG simulations. (D) Selected Martini CGMD trajectories showing the highly dynamic IDRs of p53 prior to binding ASPP. During the 4 μ long Martini CG simulations, p53's IDRs sampled various conformations before binding ASPP.

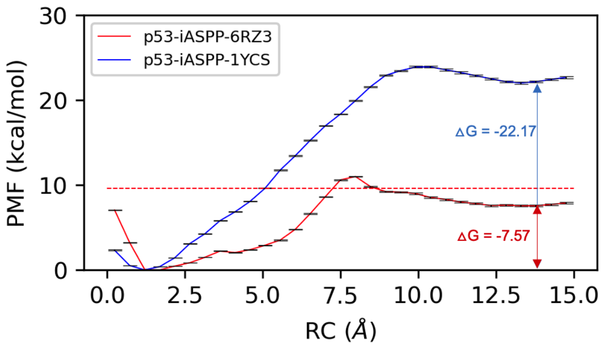

Figure S4: PMFs of iASPP-p53 dissociation starting from the crystallography-determined binding mode (PDB 6RZ3), and from the PDB 1YCS binding mode (assuming iASPP and p53 can bind in this mode).

S2 Selecting representative p53-ASPP complexes from Martini CGMD sampled protein-protein complexes

A center of mass (COM) distance criteria (<30 Å) and RMSD criteria (<50 Å, with respect to PDB 1YCS) were applied on the accumulated Martini trajectories to filter out frames that do not have p53 and ASPP contacted. The surviving trajectory frames were then aligned on $p53_{DBD}$ and were subject to following processes: ASPP protein densities around $p53_{DBD}$ were calculated using the *grid* command from the CPPTRAJ program. Regions have high relative density (> 0.6) were identified as the most probable binding patterns. The COM of ASPP was drawn around p53 for each frame. For those frames their COMs are located within the high density regions, they are identified as candidates. 20 frames were randomly picked out from the candidate pool.

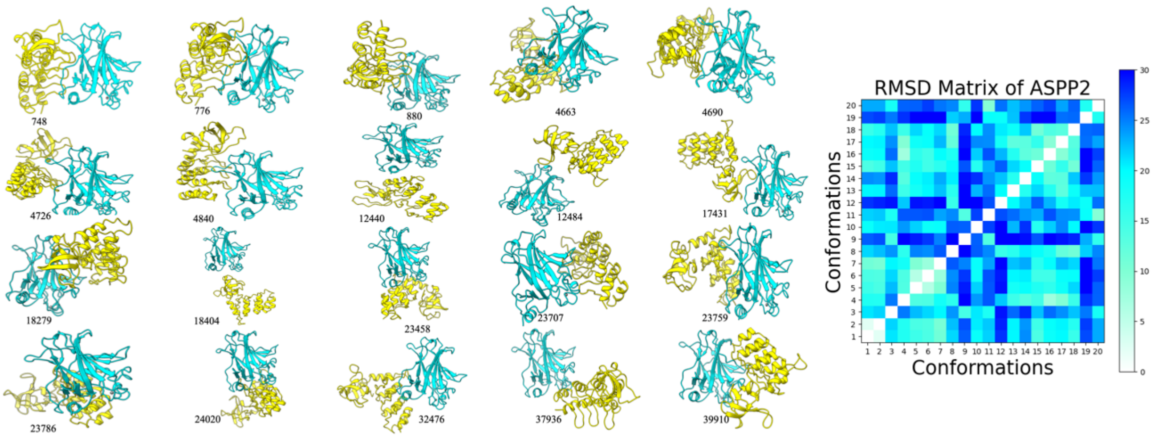

Figure S5: 20 representative $p53_{DBD}$-ASPP2 complexes sampled by Martini CGMD. The pair-wise RMSDs of ASPP2 (after aligning on p53) were shown on the right to illustrate the structural difference of ASPP2.

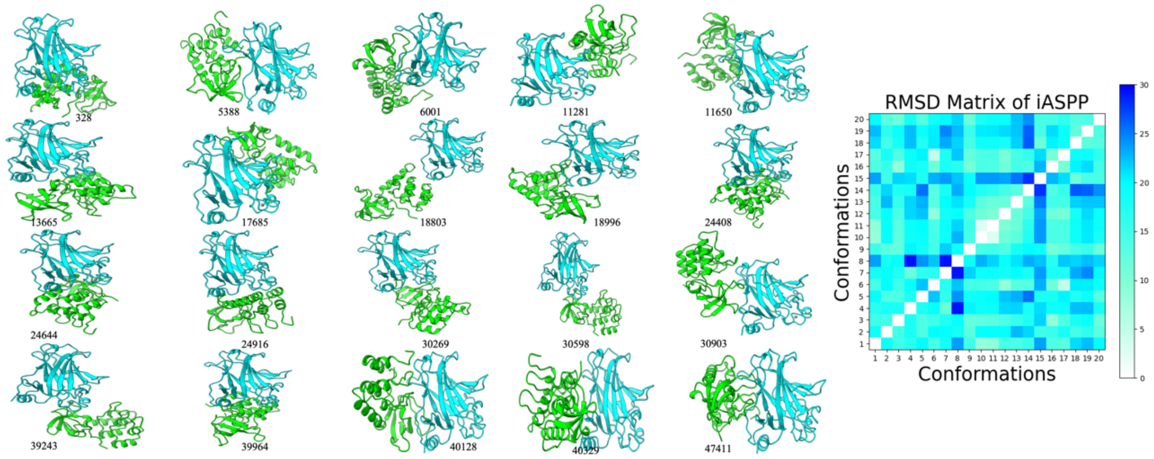

Figure S6: 20 representative $p53_{DBD}$-iASPP complexes sampled by Martini CGMD. The pair-wise RMSDs of iASPP (after aligning on p53) were shown on the right to illustrate the structural difference of iASPP.

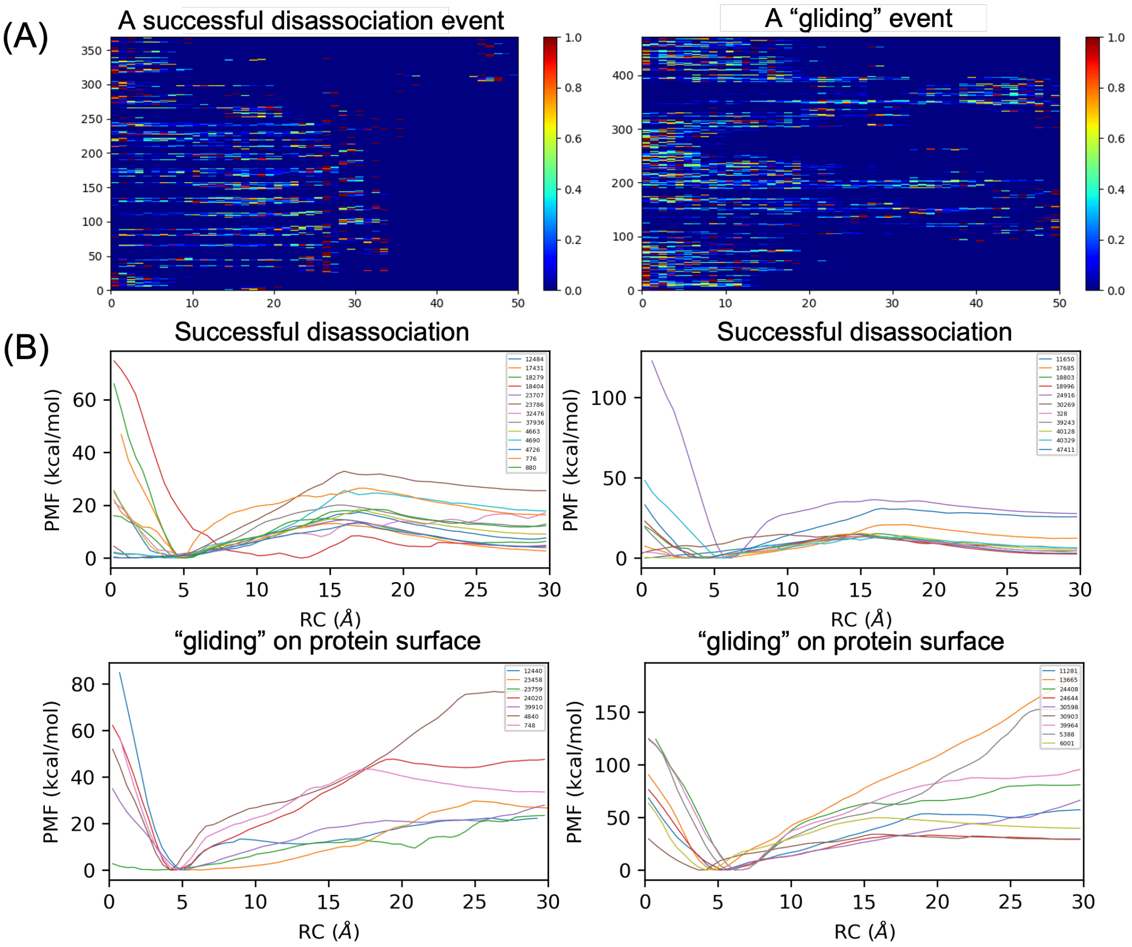

Figure S7: (A) Umbrella sampling using COM-COM distance as CV can lead to protein gliding on another protein's surface instead of driving complex directly disassociate. We show that during the umbrella sampling, a successful disassociation has inter-protein contacts smoothly disappear as window number increases, indicating a clean one-way disassociation, while for a ”gliding” event, newly formed inter-protein contacts are keep emerging as window number increases. (B-C) For the 20 representative Martini CGMD-sampled complexes for each ASPP protein, the PMF curves for successful disassociations (n=13/20 for ASPP2, and n=11/20 for iASPP), and for ”glidings” are given.
