# Supplementary material for: Reconciling ASPP-p53 binding mode discrepancies through an ensemble binding framework that bridges crystallography and NMR data
Source: PLoS Comput Biol. 2024 Feb 7;20(2):e1011519. doi: 10.1371/journal.pcbi.1011519 (PMC10878502; doi:10.1371/journal.pcbi.1011519)
Supplement: Attachment — Submitted filename: R2_response.pdf [file pcbi.1011519.s010.pdf]

0.1 Peer Reviewer #1:

Issues:

1. *Page 12, line 523: Though it is arguably a qualitative question, I do not see that the RMSD and radius of gyration of the IDRs are approaching a steady value over the 500 ns conventional MD simulations. It is hard to assess this with only a single simulation performed. Since the goal of these simulations was just to generate a reasonable starting structure for these IDRs, I suggest the authors remove this statement.*

   - **Response**
     Thanks for the comments. We reviewed Fig. S3 and agree with the reviewer that stating the RoG and RMSD of both p53's IDRs are approaching steady values over the 500 ns cMD is not strictly accurate, especially for the Pro-domain. We have removed the statement in the text, and emphasize instead that reasonable starting structures were achieved as evidenced by Fig. S3B. **See tag R1Q1 on Page 8, Line 250**

2. *Figure 3A: The values reported are normalized to the highest value observed, but all the normalized values are below 0.25. I would expect to see a value of 1 as the maximum value observed for both the chemical shift change value and the contact frequency. This should be clarified. Possibly the chemical shift change was not normalized and the contact frequency was normalized to have the same maximum value as the chemical shift change.*

   - **Response**
     We sincerely appreciate the reviewer's thorough examination of our work. In both our initial draft and revision 1, our focus was on comparing the relative scales of NMR and Martini CG data, and overlooked the details regarding normalization. The reviewer's speculation is indeed what we did, namely, NNR data were not normalized while the contact frequency data were normalized with respect to a chosen-constant to have maximum value as NMR data. We have redone the normalization of contact frequency to the highest value observed, achieving a 0 to 1 range for this data. This does not affect the relative scales but can reduce confusion.

3. *Figure 3A: The left axis should be labeled "chemical shift change" or "normalized chemical shift change"*

   - **Response**
     Done

4. *Figure 3A: Although the NMR data does not perfectly match the Martini simulation data, this could be explained by the idea raised in the discussion that the Martini ensemble represents the encounter complex rather the fully bound state. This could be addressed in the discussion.*

   - **Response**
     This is a fresh angle to possibly explain the moderate mismatch between Martini and NMR data. We added the following: Additionally, treating the Martini CGMD sampled complexes as encounter complexes can possibly explain the moderate mismatch between Martini and NMR data in Fig. 3A, as those simulated complexes need refold to reach the NMR-detected complexes which are generally treated as fully bound complexes. **See tag R1Q4 on Page 19, Line 578**

5. *Page 17, lines 693-698: I suggest the authors rephrase to say that iASPP has a wider range of affinity binders than ASPP2 rather than mentioning a distribution or affinity spectrum since that is no longer shown in the figure.*

   - **Response**
     Thanks for the helpful suggestion, we have revised the text accordingly. **See tag R1Q5 on Page 13, Line 427**

6. *Page 17: It would be interesting to comment more on the fact that the 1YCS binding pose is a stronger binder than the 6RZ3 binding pose or the Martini CG ensemble for iASPP. Does this indicate that the 1YCS binding pose is more like the fully bound state and the Martini CG binding poses represent the encounter complex.*

   - **Response**
     This is an intriguing comment. The implication of this statement is to use thermodynamic stability to classify binding complexes into encounter complexes and fully bound states, assuming the latter being more thermodynamically stable. However, conducting quantitative thermodynamic characterizations for encounter complexes can be challenging. These complexes are typically stabilized by case-dependent, non-specific, and transient electrostatic and hydrophobic interactions that are difficult to characterize [Ref55]. Nevertheless, it is widely accepted that fully bound states are thermodynamically more stable than encounter complexes, and the protein-protein binding free energy landscape is funnel-like [Ref52]. By employing a multi-funnel landscape model that represents different biological relevant bound states, we can explain the higher affinity of the 1YCS binding

mode compared to the Martini sample-average affinity or the PDB 6RZ3 mode. This involves treating the 1YCS mode as a biologically relevant mode situated at the funnel bottom, while our Martini samples contain encounter complexes (as shown in Fig 6) that average down the binding affinities. The weaker 6RZ3 binding mode can be speculated as a specific encounter complex to another funnel (rather than 1YCS) but is trapped in the crystal environment. **See tag R1Q6 on Page 19, Line 583**

7. *Fig 4D: This figure is much clearer than the previous figure. It would be nice to also include the p-values comparing the experimental data to the calculated delta Gs from the crystal structures.*
   - **Response**
     Done
8. *Page 19, lines 739-741: The discussion about kinetics is still a bit confusing in the text. I suggest the authors use wording similar to their response to my comment 11 to explain that the adding IDRs does not cause a difference in kinetics between ASPP2 and iASPP.*
   - **Response**
     Thanks for the suggestion, we have revised accordingly.
9. *Figure 5C: It would be nice to include the crystal structures in the sketch-map for reference.*
   - **Response**
     Done
10. *Page 21: I'm confused about how there seems to be one primary binding mode for iASPP in figure 5C but in figure 3C there are multiple binding modes*
    - **Response**
      Thank you for the question. In Fig. 3C, we computed the atom density of the entire p53, including the highly dynamic Pro and linker IDRs. Our objective was to demonstrate the proximity of the linkers to iASPP's RT loop in space. In the sketch-map (Fig. 5C), we encoded binding conformations using distances between p53's rigid elements (α helices, β sheets) and ASPP. We adjusted the color scale in Fig. 3C to emphasize high-density modes (> 0.8) and illustrated that there are actually few denser modes. This color adjustment does not affect similar density plots in Fig. 4C, where no p53 IDRs were included, suggesting that the dynamic IDRs contribute to the spreading of the density cloud. Another factor that might contribute to the mismatch between Cartesian space representation (Fig. 3C) and sketch-map's low-dimensionality projections (Fig. 5C) is the sketch-map's intrinsic preference for reconstructing connectivities among closer data pairs. Its ability of recapturing far-away data pairs fades due to its usage of a sigmoid function [Ref36,37]. This may overdampen the presentation of less-similar binding modes after projection. We have added comments in several parts to reflect these points. **See tag R1Q10a on Page 11, Line 382** and **See tag R1Q10b on Page 9, Line 313**
11. *Page 22-23: It would be helpful to explain the refolding idea more, including how the authors determined when refolding occurred exactly. I am not sure if it was determined by when the RMSD plateaued.*
    - **Response**
      Thanks for your interest. We have added the following in the discussion to elaborate the refolding idea. Although an objective and uniform structural definition of an encounter complex is lacking, one characteristic of encounter complexes is that they undergo structural refolding to eventually form fully bound (native) states [Ref52]. Therefore, in our Martini CGMD simulations, we assessed whether the sampled complexes exhibited or are undergoing structural reorganizations. We used RMSD to measure structural reorganization in the complexes relative to a particular state, specifically the complex at the initial point of contact. An increasing and continuously changing RMSD indicates ongoing structural refolding, while a plateaued RMSD suggests that the refolding has completed. Conversely, a consistently small RMSD suggests no appreciable refolding is occurring. **See tag R1Q11 on Page 18, Line 554**
12. *Page 23: On line 843 the authors mention "portions of simulated complexes" and line 844-845 "subsets of the encounter complexes." It is not clear if these are referring to an ensemble of encounter complexes or literally part of the complex structure.*
    - **Response**
      Thanks for pointing this out. We have changed the "portions of simulated complexes" to "portions of encounter complexes" as we have provided the context of treating Martini CG complexes as encounter complexes, and to be consistent with subsequent references.
